# Cellular Functional Analyses of *ARX* Variants Reveal New Insights Into Genotype–Phenotype Correlations in Neurodevelopmental Disorders Among Male and Female Patients

**DOI:** 10.1155/humu/4732622

**Published:** 2026-04-07

**Authors:** Rasha Faraj, Audrey Farrugia, Anna C. E. Hurst, Pierre Conan, Jennifer Martin, Audrey Schalk, Sylvia Redon, Aline Dubos, Mathilde Gras, Aurore Curie, Cécile Voisset, Gaëlle Friocourt

**Affiliations:** ^1^ UMR 1101, LaTIM, Inserm, IMT-A, Univ Brest, Brest, France, inserm.fr; ^2^ UMR 1078, GGB, Inserm, EFS, Univ Brest, Brest, France, inserm.fr; ^3^ Institut de Médecine Légale de Strasbourg, Fédération de Médecine Translationnelle de Strasbourg (FMTS), Université de Strasbourg, Strasbourg, France, unistra.fr; ^4^ Institut de Génétique et de Biologie Moléculaire et Cellulaire (IGBMC), CNRS UMR7104 INSERM U1258, Université de Strasbourg, Illkirch, France, unistra.fr; ^5^ Department of Genetics, University of Alabama, Birmingham, USA, ua.edu; ^6^ Laboratoire de Diagnostic Génétique, Nouvel Hôpital Civil, Hôpitaux Universitaires de Strasbourg, Strasbourg, France, chru-strasbourg.fr; ^7^ Service de Génétique Médicale et Biologie de la Reproduction, CHRU de Brest, Brest, France; ^8^ Reference Centre of Rare Disease With Intellectual Disability and Multiple Disabilities, CHRU de Brest, Brest, France; ^9^ Department of Clinical Genetics, APHP Sorbonne Université, University Hospital Pitié Salpêtrière, Paris, France; ^10^ Child Neurology Department and Reference Centre of Rare Disease With Intellectual Disability, Hospices Civils de Lyon, Lyon University Hospital, Lyon, France, chu-lyon.fr; ^11^ CNRS UMR5292, INSERM U1028, Lyon Neuroscience Research Centre, Lyon, France, inserm.fr; ^12^ Lyon University, Lyon, France, universite-lyon.fr

**Keywords:** cerebral palsy, dystonia, epileptic encephalopathy, intellectual deficiency, interneuronopathy, neurodevelopment, spastic quadriplegia

## Abstract

Neurodevelopmental disorders (NDDs) encompass a wide range of conditions often linked to genetic causes, with mutations in the X‐linked *ARX* gene representing a recurrent contributor. *ARX* encodes a transcription factor critical for GABAergic neuron development and functioning, regulating the expression of key neurodevelopmental target genes. Variants in *ARX* result in a wide clinical spectrum, ranging from asymptomatic female carriers to severe developmental syndromes in both sexes. This study investigates the functional impact of 16 *ARX* variants, including known pathogenic, likely pathogenic, and novel de novo variants, some associated with atypical presentations such as sudden infant death. Using transient expression in N2a neuroblastoma cells, we employed a comprehensive functional approach—including luciferase reporter assays, Western blotting, immunofluorescence, and RT‐qPCR—to assess protein expression levels, subcellular localization, their interaction with known corepressors (TLE1 and CtBP1), and their transcriptional activity on selected *ARX*‐known targets. Our results demonstrate that all tested variants disrupt normal *ARX* transcriptional function, with several also altering protein localization or expression. Remarkably, a subset of variants exhibited dominant‐negative effects, offering a compelling explanation for unexpectedly severe phenotypes in female patients, likely exacerbated by skewed X‐inactivation and other modifying factors. By integrating experimental data with a literature‐based review of published *ARX* variants, we provide refined genotype–phenotype correlations, highlighting the importance of mutation type, positional context within key *ARX* functional domains, and patient sex. Altogether, this study advances our understanding of the molecular mechanisms driving *ARX*‐related NDDs, emphasizing the importance of functional testing for accurate variant interpretation and paving the way toward informed genetic counseling and potential therapeutic development.

## 1. Introduction

Neurodevelopmental disorders (NDDs) encompass a broad spectrum of conditions characterized by significant disruptions in the growth and development of the brain and central nervous system. These disorders typically emerge in childhood and often lead to lifelong challenges, profoundly affecting cognitive abilities, motor functions, communication skills, and behavioral patterns. NDDs include conditions such as intellectual disability (ID), autism spectrum disorder (ASD), epilepsy, and developmental motor impairments, among others. The etiology of NDDs is complex and multifactorial, involving an intricate interplay of genetic, epigenetic, and environmental factors. Notably, there is an increasing evidence that genetic mutations, particularly those affecting genes involved in early brain development and neuronal differentiation, play a substantial role in their pathogenesis [1].

Despite comprising only about 5% of the human genome, the X‐chromosome harbors mutations in approximately 20% of its genes that are linked to NDDs, with over 140 X‐linked genes robustly associated with ID [2, 3]. Among these, the Aristaless‐related homeobox gene (*ARX*, MIM# 300382) stands out as a key regulator of neurodevelopment and is among the most frequently mutated genes associated with X‐linked forms of ID [4, 5].

**Figure 1:**
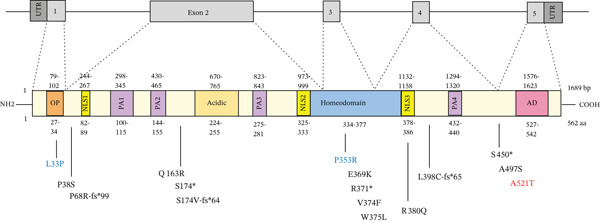
Distribution of the *ARX-*studied variants along the protein sequence. Schematic representation of *ARX* gene and its five exons, and the distribution of the studied variants along the *ARX* protein sequence. The protein domains and important functional regions of *ARX* including the OP domain, three nuclear localization signals (NLS), four poly‐alanine repeats (PA), an acidic domain (Acidic), a Prd‐like homeodomain and a C‐terminal Aristaless domain (AD) are depicted. Above the representation of the protein are indicated the nucleotide numbers of the different domains. Below the representation of the protein are indicated the corresponding amino acids. The localization of variants studied here is indicated, p.L33P and p.P353R being included as positive controls in some of the experiments, and the variant p.A521T, which has been identified in control subjects, was used as a negative control.

The *ARX* gene, located on the short arm of the X‐chromosome at position Xp22.13, consists of five exons spanning approximately 12.5 kb of genomic DNA and encodes a 562‐amino‐acid protein [4, 6, 7] (Figure 1). *ARX* belongs to the Group II Aristaless‐related protein family, whose members are predominantly expressed in the central and peripheral nervous system. The *ARX*‐encoded protein functions as a key transcription factor and belongs to the Aristaless‐related group within the paired (Prd) class of homeodomain proteins. It contains several functional domains, including an N‐terminal octapeptide (OP) domain, an acidic domain, a homeodomain, four polyalanine (PA) tracts, three nuclear localization sequence motifs (NLS), and a C‐terminal Aristaless domain (AD) (Figure 1) [8, 9]. *ARX* expression is primarily observed in the embryonic brain, endocrine pancreas, and testes [10]. However, its activity also extends to the adult brain, heart, skeletal muscle, and liver, underscoring its diverse roles in both developmental and physiological processes [11]*. ARX* plays a key role in neurodevelopment, particularly in the differentiation and migration of GABAergic interneurons within the cerebral cortex and ventral forebrain [6, 12–14]. This essential function has led to the use of the term “interneuronopathy” to describe syndromes associated with *ARX* mutations [15]. As a transcription factor, *ARX* binds DNA via its homeodomain and functions as both a transcriptional activator and repressor [11, 16], regulating the expression of multiple downstream target genes involved in brain development and function.

Variants in the *ARX* gene are associated with a broad spectrum of NDDs. First identified in males, the associated phenotype in this group is characterized by a broad spectrum of features: ID is a consistent hallmark, whereas other features, such as epilepsy, abnormal genitalia, and brain malformations, vary widely [17]. The phenotypic spectrum ranges from severe syndromes characterized by brain structural abnormalities, such as X‐linked lissencephaly with abnormal genitalia (XLAG), hydranencephaly, and agenesis of the corpus callosum (ACC; MIM 300004, also known as Proud syndrome), to milder, less life‐threatening phenotypes involving motor impairments and epilepsy without overt brain structural malformations [9, 18, 19]. Among the hallmark features of *ARX*‐related disorders, motor impairments, including dystonia and developmental limb kinetic apraxia (LKA), are key manifestations [20]. These are linked to dysfunctions in cortical and subcortical networks, particularly in the basal ganglia and motor cortex [20–22]. These motor deficits may appear as early as infancy and often persist throughout life, with a significant impact on patients′ quality of life. These impairments have been observed not only in patients [20, 23, 24], but are also recapitulated in mouse models, highlighting a crucial role for *ARX* in motor circuit development [22, 25–27].

Multiple mutations and variants in the *ARX* gene have been extensively reported in male patients [9, 19, 28]. In contrast, until recently, females carrying pathogenic *ARX* variants were largely considered as asymptomatic or with only mild phenotypes [29]. However, recent reports describe novel *ARX* variants in women, associated with a broader spectrum of clinical manifestations, ranging from asymptomatic to ID, ASD, and epilepsy, and often accompanied by abnormalities of the corpus callosum [24, 30]. These recent studies, as well as the increasing description of new *ARX* variants in males, have expanded the known phenotypic spectrum and underscored the need for further investigation into the functional consequences of these mutations. To address these knowledge gaps, therefore, the aim of this study is to functionally characterize the impact of both novel and previously described *ARX* variants (Figure 1), with particular emphasis on their effects in female carriers. This work is aimed at providing deeper insights into the molecular consequences of these variants and to explore potential genotype‐phenotype correlations, thereby contributing to the understanding of the mechanisms underlying phenotypic variability between males and females.

## 2. Material and Methods

### 2.1. Patient Information

Clinical information, laboratory findings, imaging, neuropathological data, and genetic testing for the individuals listed in Table 1 were collected and evaluated as part of standard clinical care. Genetic results were matched through Genematcher and shared upon written informed consent obtained from all individuals or their parents. All variants included in this study and the patient’s phenotype are described in Table 1 and in supporting data for the two patients (Patients 8 and 15; Table S1) that had not been previously described.

**Table 1:** List of variants used in functional studies. Note that the variant p.A521T has also been identified in several control subjects and may thus not be pathogenic.

	Mutation (DNA) (NM_139058)	Mutation (protein)	Familial or de novo	Phenotype	Reference
1	c.98T>C	p.L33P	Nine male patients across two generations (family P8 = MRX54)	X‐linked moderate to profound ID, two had epilepsy, one had aggressive behavior	Ben Jemaa, Am J Med Genet 1999; Bienvenu, HMG 2002
2	c.112C>T	p.P38S	A male patient (P109)	Severe language delay, obesity and autistic behavior	Poirier, Neurogenetics 2006
3	c.201_204del	p.P68Rfs∗99	A female patient (Case 3) de novo	Pharmacosensitive focal seizures, mild ID, partial ACC	Gras, J Med Genet 2023
4	c.488A>G	p.Q163R	Six male patients in a same family (T4) de novo	X‐linked moderate to profound ID, cerebellar symptoms in 2/6 patients, 5/6 patients had epilepsy, 3/6 patients were irritable and aggressive	Bienvenu, HMG 2002
5	c.518dupA	p.S174Vfs∗64	A female patient (Case 9) de novo	Moderate ID, ASD, complete ACC, pharmacosensitive epilepsy, orolingual dyspraxia and fine motor skills impaired	Gras, J Med Genet 2023
6	c.521C>A	p.S174∗	A female patient (Case 5) de novo	Moderate ID, ASD + ADHD, complete ACC, no epilepsy, poor coordination	Gras, J Med Genet 2023
7	c.1058C>G	p.P353R	A male patient (LR02‐162) de novo	X‐linked lissencephaly with abnormal genitalia	Kato, Hum Mut 2004
8	c.1105G>A	p.E369K	A 12 mo male patient de novo	Microcephaly, seizures (initially infantile spasms but emerging into tonic‐clonic and a Lennox‐Gastaut pattern), ACC with subtle loss of white matter volume in left cerebral hemisphere, hypotonia, stridor, and dysmorphic features (bitemporal narrowing, long palpebral fissures, epicanthal folds, short columella, long philtrum, tented upper lip with upturned vermillion border)	This study
9	c.1111C>T	p.R371∗	A female patient (Case 2) de novo	Pharmacoresistant generalized seizures + febrile seizures, DEE, complete ACC, severe ID, dystonia left side	Gras, J Med Genet 2023
10	c.1120G>T	p. V374F	A female patient (Case 6) de novo	DEE, pharmacoresistant seizures, partial ACC, severe ID, hypotonia	Gras, J Med Genet 2023
11	c.1124G>T	p.W375L	A female patient (Case 8) de novo	Severe ID, stereotypie, DEE, complete ACC, gyration abnormalities, pharmacosensitive seizures, unable to walk	Gras, J Med Genet 2023
12	c.1139G>A	p.R380Q	A female patient (Case 4) de novo	DEE, pharmaco‐resistant seizures, complete ACC, severe ID, gyration abnormalities	Gras, J Med Genet 2023
13	c.1191del	p.L398Cfs∗65	A female patient (Case 7) de novo	Partial ACC, ASD, gestual dyspraxia	Gras, J Med Genet 2023
14	c.1349C>A	p.S450∗	A female patient (Case 1) de novo	Pharmacoresistant epilepsy, ISSX, DEE, complete ACC, severe ID, pyramidal syndrome	Gras, J Med Genet 2023
15	c.1489G>T	p.A497S	A boy of 2 months and a half mosaic de novo	Sudden infant death (see supporting data for clinical description)	This study
16	c.1561G>A	p.A521T	Predicted as a VUS by several prediction softwares	Variant found in 1.8e‐05 of 1171641 control chromosomes, including six hemizygotes (GnomAD)	

Abbreviations: ACC, agenesis of the corpus callosum; ADHD, attention‐deficit/hyperactivity disorder; ASD, autism spectrum disorder; DEE, developmental epileptic encephalopathy; ID, intellectual deficiency; ISSX, X‐linked infantile spasms syndrome; VUS, variant of unknown significance.

### 2.2. Expression Plasmids Used for the Expression of *ARX*, TLE1, and CtBP1

The QuikChange site‐directed mutagenesis kit (Stratagene, La Jolla, California, United States) was used to introduce single nucleotide substitution mutations into the human wild‐type (WT) *ARX* cDNA, subcloned in the Gateway‐compatible pCMV‐myc vector (BD‐Biosciences) with an N‐terminal in‐frame myc‐tag (Invitrogen), following the manufacturer′s instructions. This plasmid was a kind gift from C. Shoubridge. Primers used to generate the different mutations are listed in Table S2.

To assess the effect of corepressors in luciferase experiments, TLE1 and CtBP1 human cDNAs were amplified from HepG2 cells and subcloned into the EGFP‐C1 expression vector (Addgene, #58457) for TLE1 and into the p3xFLAG‐CMV plasmid (Sigma‐Aldrich, #E4151) for CtBP1, using primers listed in the supporting data (hTLE1‐Xho‐GFP‐F and hTLE1‐Xho‐GFP‐R for TLE1; hCtBP1‐Hind‐FLAG‐F and hCtBP1‐EcoRI‐FLAG‐R for CtBP1). All generated plasmids were verified by enzyme digestion and Sanger sequencing.

### 2.3. Cell Culture

Neuro2a (N2a) mouse neuroblastoma cells (IFO50081) were obtained from the Japanese Collection of Research Bioresources (http://cellbank.nibio.go.jp/). Cells were grown in Dulbecco′s modified Eagle Medium (DMEM F12, a 1:1 mixture of DMEM and Ham′s F12 media), which was supplemented with 10% fetal bovine serum, 0.1‐mM nonessential amino acids (Gibco), and 100‐U/mL penicillin/streptomycin (Invitrogen). HEK293T human embryonic kidney cells were obtained from ATCC. These cells were cultured in Dulbecco′s modified Eagle medium (DMEM, Gibco) supplemented with 10% fetal bovine serum, 0.1‐mM nonessential amino acids, and 100‐U/mL penicillin/streptomycin. Cells were grown at 37°C with a 5% CO_2_ atmosphere in a humidified incubator and transiently transfected with the myc‐tagged vector expressing *ARX* mutant proteins using either Lipofectamine 2000 (Invitrogen) or jetOPTIMUS (Polyplus transfection, Illkirch, France) transfection reagents according to the manufacturer′s guidelines.

### 2.4. Western Blot

N2a cells were seeded at 400,000 cells per well in six‐well plates 24 h before transfection. Cells were then transfected with 1.5 *μ*g of either the empty myc vector or the myc vector containing WT or mutant *ARX* cDNA using Lipofectamine 2000 transfection reagent following the manufacturer′s instructions. After 24 h of transfection, cells were harvested in the following buffer: 150‐mM NaCl, 1% Igepal, 50‐mM Tris–HCl pH 7.4 with a protease inhibitor cocktail (Roche). Cell lysis was then performed by six cycles of vigorous vortexing and freeze‐thawing. The protein amount in the supernatants was evaluated by the classical Bradford assay. A total of 25 *μ*g of protein for each sample were then loaded into 10% NuPAGE Bis‐Tris gels (precast NuPAGE, Invitrogen), and transferred onto 0.45‐*μ*m nitrocellulose membranes (Cytiva, Velizy‐Villacoublay, France). Membranes were blocked for 1 h at room temperature in 1X PBS (phosphate‐buffered saline) containing 0.1% Igepal and 5% fat‐free milk and then incubated overnight at 4°C with the following primary antibodies: rabbit anti‐*ARX* antibody (directed against amino acids 310–396 [10], 1:1000), mouse anti‐myc antibody (Sigma‐Aldrich, #M5546, 1:5000) and mouse monoclonal anti–*α*‐tubulin antibody (Sigma‐Aldrich, #T6793, 1:10,000). The following day, membranes were washed with fresh 1X PBS with 0.1% Igepal and incubated for 45 min with secondary antibodies: goat antirabbit (Thermo Scientific, #31460, 1:5000) for the anti‐ARX antibody, and goat antimouse (Abcam, #ab6789, 1:3000) for the anti‐myc and anti–*α*‐tubulin antibodies, conjugated to horseradish peroxidase. Images were then acquired by enhanced chemiluminescence (ECL, Cytiva) using a Fusion FX‐7 imaging system from Vilber.

### 2.5. Luciferase Assays

DNA genomic fragments corresponding to promoter regions of *Lmo1*, *Calb2*, or *Cdh2* genes were amplified by PCR from mouse genomic DNA and subsequently cloned into the pGL4.23 firefly luciferase vector (Promega, E8411) using the In‐Fusion cloning strategy (Clontech) and primers listed in the supporting data (mLmo1‐F and mLmo1‐R for *Lmo1;* mCalb2‐F and mCalb2‐R for *Calb2*; mCdh2‐F and mCdh2‐R for *Cdh2*).

N2a cells were seeded at 120,000 cells per well in 24‐well plates. The N2a cells were transfected 24 h after plating with 500 ng of *Lmo1*, *Calb2*, or *Cdh2* promoters cloned into the luciferase reporter plasmid pGL4.23 expressing the *firefly* luciferase (Promega), 200 ng of ARX‐expressing myc vector or empty vector and 100 ng of pGL4.74 vector expressing the control *Renilla* luciferase (Promega) using Lipofectamine 2000. At 48 h after transfection, cell lysis and measurement of *firefly* and *Renilla* luciferase activity were performed using the Dual‐Glo Luciferase Assay System (Promega) according to the manufacturer′s instructions on a Varioskan LUX Multimode Microplate Reader (Thermo Scientific). The *firefly* luciferase activity was normalized according to the corresponding *Renilla* luciferase activity to correct for transfection efficiency, and luciferase activity was reported as the mean relative to the results obtained with the empty expression vector. Each transfection was performed in triplicate and the experiment was conducted a minimum of three times. Significant differences were assessed by one‐way ANOVA with Dunnett′s post hoc statistical test using the Prism software from GraphPad (La Jolla, United States).

### 2.6. Immunofluorescence Assays

N2a cells were seeded at 75,000 cells per well in Nunc Lab‐Tek II Chamber Slide (Thermo Scientific). The N2a cells were transfected 24 h after plating with 500 ng of each construct using jetOPTIMUS transfection reagent according to the manufacturer′s instructions. At 24 h after transfection, cells were washed once with 1X PBS (pH 7.4) and fixed with 4% paraformaldehyde (Sigma‐Aldrich, United States) for 20 min at room temperature. After fixation, cells were washed with 1X PBS, and then nonspecific binding sites were blocked by incubating cells in a blocking solution of 10% normal goat serum and 0.2% Triton X‐100 (Sigma‐Aldrich, United States) for 1 h at room temperature. After blocking, the cells were incubated with anti‐myc and anti‐*ARX* (same antibodies as those used for Western blotting) primary antibodies diluted 1:500 in a 1:10 blocking solution at 4°C overnight. The next day, after three washes with 1X PBS, the cells were incubated with fluorophore‐conjugated secondary antibodies Alexa Fluor 568 antimouse (Invitrogen) or Alexa Fluor 488 antirabbit (Invitrogen) diluted at 1:400 in 1:10 blocking solution for 2 h at room temperature in the dark. To visualize nuclei, cells were stained with 4 ^′^,6‐diamidino‐2‐phenylindole (VECTASHIELD Antifade Mounting Medium with DAPI, 1.5 *μ*g/mL; Vector laboratories) for 5 min at room temperature in the dark, and then slides were mounted and imaged using a fluorescence microscope (Axio Imager M2, Carl Zeiss, Germany). Phase contrast and fluorescent images were collected with the 20× and 40× objectives and image analysis was performed with the Zen 2.3 SP1 software (Carl‐Zeiss Microcopy GmbH, Jena, Germany).

### 2.7. RT‐qPCR

N2a cells were seeded at 400,000 cells per well in six‐well plates 24 h before transfection. Cells were then transfected with 1.5 *μ*g of either the empty myc vector or the myc vector containing *ARX* cDNA constructs using Lipofectamine 2000 transfection reagent, following the manufacturer′s instructions. At 48 h after transfection, RNA was isolated using the NucleoSpin RNA Plus mini kit (Macherey‐Nagel), and RNA concentration was measured using a NanoDrop One/OneC Microvolume UV‐Vis spectrophotometer. Reverse transcription was performed using the LunaScript RT SuperMix Kit (NEB#E3010), following the manufacturer′s instructions. Real‐time PCR was performed on an Azure Cielo real‐time PCR system using ONEGreen FAST qPCR Premix (Ozyme, #OZTA008‐40 and OZYA008‐200XL). Expression levels of *Lmo1*, *olfactomedin-1* (*Olfm1*), and *L1cam* ARX target genes were normalized across samples using the *β-actin* housekeeping gene. The primers used (annotated qF or qR) are listed in the supporting data. Data analysis was done by the 2^–*ΔΔ*Ct^ method, and relative expression was calculated using the empty myc‐tagged vector condition as the reference sample (expression = 1). Significant differences were assessed by the ordinary one‐way ANOVA test or Student′s t test.

### 2.8. Statistical Analysis

Statistical analysis was performed using the GraphPad Prism software (San Diego, California). Presented results represent data from a minimum of three independent experiments.

## 3. Results

### 3.1. Expression and Stability Profiles of *ARX* Variant Proteins

One of the objectives of this study is to present simple functional tests that can be used to decipher the functional consequences of *ARX* variants. So far, this type of test has predominantly been used to study *ARX* variants in the two first PA tracts PA1 and PA2 (Figure 1) [31, 32], or missense mutations located in the homeodomain [33–35]. Here, we focused on variants recently described in female patients [24] and on a few missense variants located outside canonical functional domains, such as p.Q163R or p.P38S and whose effect on *ARX* function is unclear (Figure 1). We also tested p.A521T, a variant of unknown significance (VUS) that has also been identified in control subjects (Table 1). Finally, we have included, as controls in some experiments, two missense mutations p.L33P and p.P353R, which have already been extensively characterized [16, 35–37].

Several of these variants studied here, especially the premature truncation variants (PTVs), should in theory trigger nonsense‐mediated mRNA decay (NMD) in the patient′s cells. However, as NMD is not always 100% efficient, that interindividual variability in NMD efficiency has been reported [38], and that examples of neomorphic or dominant‐negative effects of the resulting truncated proteins have been described [39, 40], it still can be useful to assess the stability, localization, and the effects of proteins potentially produced in vivo from these mRNAs. For these reasons, we also have included DNA constructs producing these truncated proteins in our assays.

First, to assess the impact of these *ARX* variants on protein expression level and stability, as well as to investigate the possibility of translational reinitiation for truncation mutations, we performed Western blot analysis in neuroblastoma N2a cells transfected with plasmids encoding the respective *ARX* myc‐tagged constructs (Figure 2). The N2a cells were chosen due to their neuronal‐like properties, ease of transfection, absence of endogenous *ARX* expression, and their previous use in functional studies of *ARX* [21, 41–43]. The protein expression level of each variant was assessed using anti‐myc (Figure 2a) or an anti‐*ARX* antibody directed against the homeodomain. Western blot analysis with the anti‐myc antibody revealed *ARX* protein bands of sizes corresponding to the expected molecular weight of each mutant (Figure 2a,b). The intensity of these bands was generally comparable with that of WT *ARX*, indicating similar protein expression levels, except for the variant p.S174∗ protein, which exhibited a noticeably lower intensity, suggesting a potential reduction in protein stability.

Figure 2Western‐blot analysis of the expression of the *ARX* protein for each studied variant. (a) Representative images of Western‐blot showing the expression of the *ARX* protein in lysates from N2a cells transfected with plasmids expressing myc–WT‐*ARX* or myc‐*ARX* variants, with an expected band for the full‐length *ARX* protein observed at approximately 64 kDa (size of the *ARX* protein with the addition of the N‐terminal myc tag). Primary antibodies used were anti‐myc and anti–*α*‐tubulin. The latter was used for normalization of protein loading. Note the decreased amount of p.S174∗ protein and the faint band of higher size (indicated by an arrow) expressed with p.S174Vfs∗64. (b) Schematic representation of expected truncated proteins obtained after transfection of nonsense and missense *ARX* variants. Abnormal C‐terminal amino acid sequences resulting from frameshift mutations are represented in gray.

**Figure:**
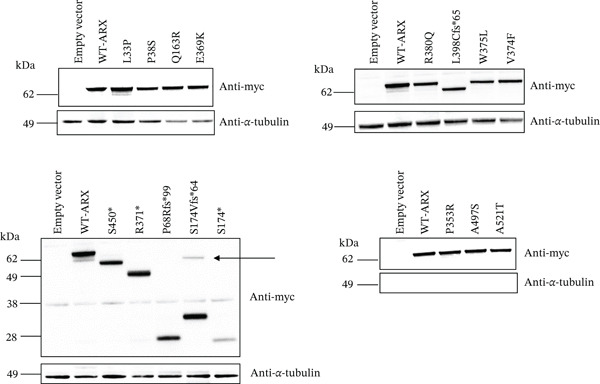
(a)

**Figure:**
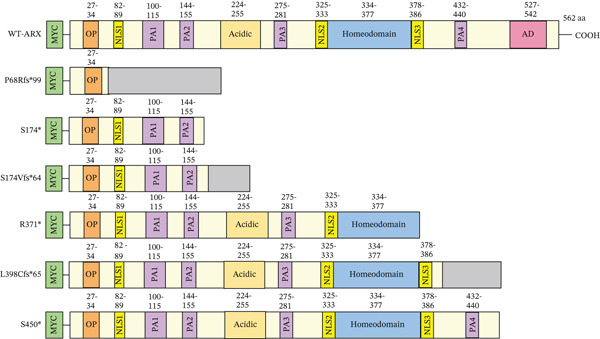
(b)

Western‐blot analysis using the *ARX* antibody confirmed previous findings [44, 45] that there is no translation reinitiation in the *ARX* protein when the premature termination codon (PTC) is located downstream amino acid 54. For example, no band other than the expected one was visible for the p.P68Rfs∗99 variant with ARX antibody (Figure S1). However, in the case of the p.S174Vfs∗64 variant, we observed the specific band at the expected size (Figure 2) but also an additional, very faint upper band, that was detected using both anti‐myc antibody and an anti‐*ARX* antibody directed against the homeodomain (Figure S1) but was not recognized with an *ARX* antibody directed against the C‐terminal part of the protein (aa 496–562), which suggests that this band likely corresponds to a protein smaller than the full‐length ARX protein. As this band was not visible with other variants, this is possibly due to RNA editing of the c.518dupA variation, which would allow, at very low level, the production of a longer ARX protein, or stop codon readthrough. The efficiency of this type of phenomenon has been reported to be influenced by the identity of the stop codon and surrounding sequence contexts [46]. Whether this full‐length protein is expressed in vivo remains uncertain due to the inaccessibility of *ARX*‐expressing tissues in the patient; however, if expressed, its level and functionality are apparently insufficient to prevent disease manifestation (see Table 1).

### 3.2. Impaired Transcriptional Repression Capacity of *ARX* Variants

Although *ARX* can function as both a repressor and an activator, its main function in the brain appears to be primarily through transcriptional repression as shown by several transcriptomic studies [42, 47, 48]. In particular, in mouse, *ARX* is a direct transcriptional repressor of *Lmo1* [42, 47, 48], *Calb2*, and *Cdh2* genes [47, 48], and the *Lmo1* promoter region has thus been successfully used in luciferase reporter assays to assess the effect of *ARX* variants located in its homeodomain or affecting the length of the PA tracts on its transcriptional activity [33–35, 45, 49]. In this study, we tested whether this assay could also be applied to assess the transcriptional repressor capacity of variants located in other domains of the *ARX* protein.

Compared to the value set for the empty myc‐vector (100%), WT‐*ARX* repressed the expression of luciferase by ~62% when transfected into N2a cells. As expected, truncation variants lacking the homeodomain (aa 334–377, Figure 2), and thus unable to bind DNA, such as p.P68Rfs∗99 and the two variants affecting residue 174, showed a severe loss‐of‐function (Figure 3a, upper panel). Similarly, the variant p.S450∗ lacking the C‐terminal part of the protein, also showed a severe loss‐of‐function effect on *Lmo1* transcriptional regulation (Figure 3a, upper panel), which confirms previous findings that the region between aa 400–495 and including the PA4 PA tract contained a strong repressor domain [16, 36]. Concerning the missense variants, several of them presented variable levels of decreased transcriptional repression activity except p.A521T, which did not show any significant effect using this assay (Figure 3a, lower panel). Interestingly, a second group of variants, that mainly include variants within or just downstream the homeodomain, exhibited aberrant transcriptional activity leading to activation rather than repression of the luciferase reporter gene usually observed with *Lmo1* promoter (Figure 3b). The only exception was the missense variant p.E369K, affecting the first residue of the Helix 3 of the homeodomain, that showed loss‐of‐function (Figure 3a, lower panel). Very similar results of the different variants were observed with *Calb2* or *Cdh2* promoter regions (data not shown), ruling out a specific behavior due to the use of *Lmo1* promoter region. However, we decided to focus our experiments on *Lmo1* promoter region since *Lmo1-*based assay was more sensitive and robust, allowing the detection of subtle variations on transcription regulation by *ARX*.

Figure 3Transcriptional repression capacity of the different *ARX* variants. Plasmids encoding WT or mutant ARX proteins were transfected in N2a cells and their capacity to repress transcriptional activity of the luciferase reporter gene under the control of *Lmo1* promoter was assessed. (a) Several truncation mutations (upper panel) as well as missense variants (lower panel) caused a severe decrease of the transcriptional repression capacity, except the variant p.A521T, which did not modify significantly *ARX* transcription activity. The variant p.L33P was used as a control. (b) Missense or truncation variants located within or downstream *ARX* homeodomain resulted in the activation instead of repression of *ARX* transcriptional capacity. (a–b) Comparison of each condition with WT‐*ARX*, one‐way ANOVA with Dunnett′s post hoc test. (c) WT‐*ARX* was cotransfected along with plasmids encoding each variant to assess the ability of WT‐*ARX* to rescue the defect of transcription of each variant. As expected, variants with loss‐of‐function (LOF) effects (represented in light gray) were rescued by WT‐*ARX* (since their effect was not statistically different from the condition empty vector + WT‐*ARX*). On the contrary, variants with apparent gain‐of‐function (GOF) effects (represented in dark gray) were not completely rescued by WT‐*ARX*, suggesting that these variants may have dominant‐negative effects on the WT‐*ARX* protein. Comparison of each cotransfection with empty Myc vector + *ARX* condition. One‐way ANOVA with Dunnett′s post hoc test: ∗∗, *p* < 0.01; ∗∗∗, *p* < 0.001, and ∗∗∗∗, *p* < 0.0001; ns: not significant. Note that half amount of each DNA variant was used in (c) compared with (b), explaining why the levels of the reporter gene activation or repression are lower in (c) compared with (b).

**Figure:**
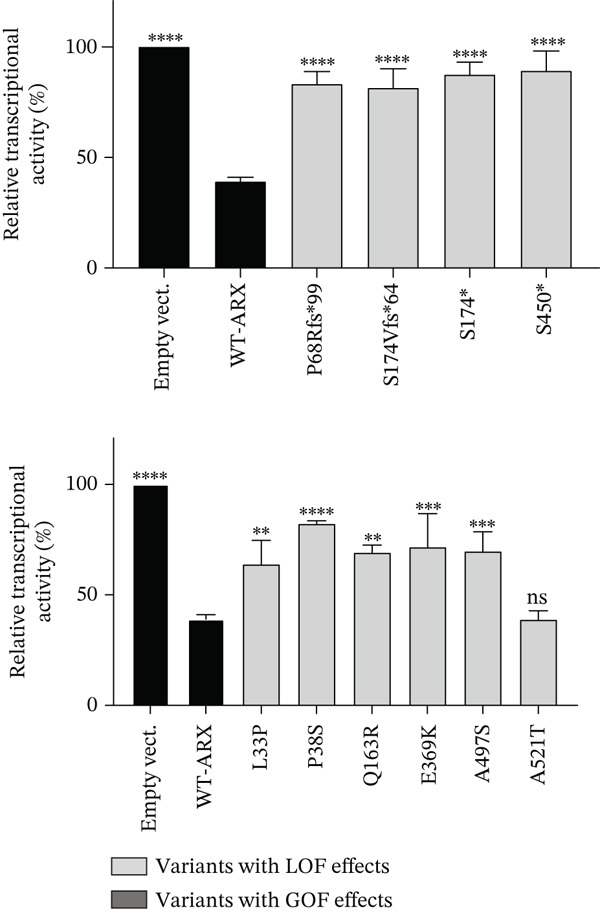
(a)

**Figure:**
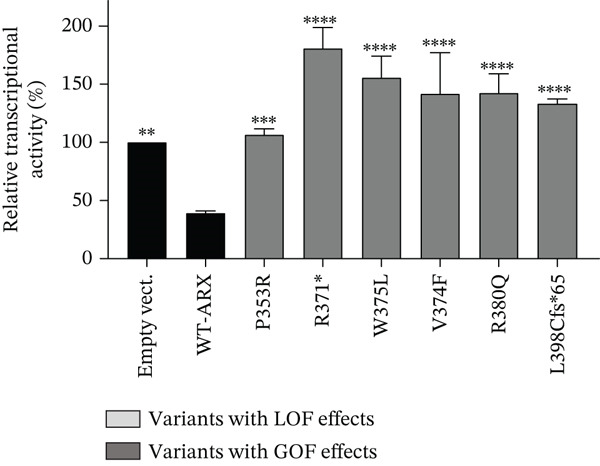
(b)

**Figure:**
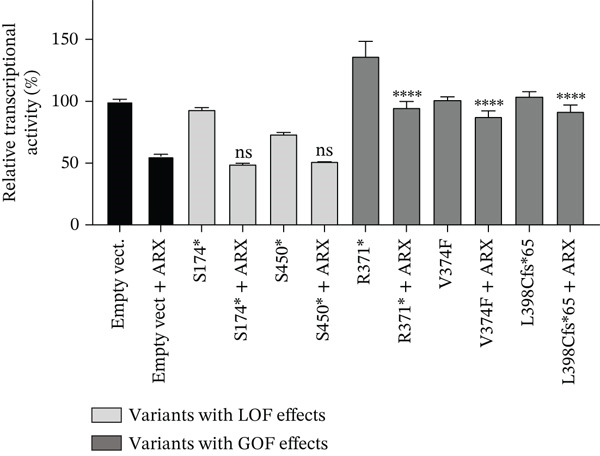
(c)

To further characterize the apparent gain‐of‐function effect of variants located within the homeodomain, we leveraged the fact that *ARX* acts as a transcriptional activator on *Lmo1* promoter region in 293T cells, probably due to interactions with different cofactors endogenously expressed in 293T versus N2a cells. As shown in Figure S2a, the loss‐of‐effect of several missense variants was confirmed, with decreased transcriptional activation compared with WT‐*ARX*. In the case of the variants that showed an apparent gain‐of‐function in N2a cells, they showed a similar increased activation compared to WT‐*ARX*. These results thus suggest a dominant‐negative effect rather than a real gain‐of‐function effect in N2a cells. Interestingly, the variant p.L398Cfs∗65 variant showed a partial loss‐of‐function effect in 293T cells (Figure S2a) contrasting with the gain‐of‐function effect observed in N2a cells (Figure 3b), suggesting that the mechanism of pathophysiology of this variant may not be the same as the variants located in the homeodomain.

To further test the hypothesis of a dominant‐negative effect, we next examined whether coexpression of WT‐*ARX* could rescue the transcriptional repression defect of the mutant proteins. As presented in Figure 3c, the expression of WT‐ARX was able to rescue the effect of all the variants presented in Figure 3a (e.g. p.S174∗ and p.S450∗ (Figure 3c) and data not shown), consistent with a loss‐of‐function mechanism. On the contrary, the coexpression of WT‐*ARX* failed to fully restore the repression of the luciferase reporter gene for the variants presented in Figure 3b (e.g. p.R371∗, p.V374F, and p.L398Cfs∗65 (Figure 3c) and data not shown). Although the luciferase expression level obtained with the latter variants was decreased by WT‐*ARX* transfection, it was significantly less repressed than the control empty vector + *ARX*, which further suggests a likely dominant‐negative effect of these variants.

### 3.3. Abnormal Subcellular Localization of Certain *ARX* Variant Proteins

**Figure 4:**
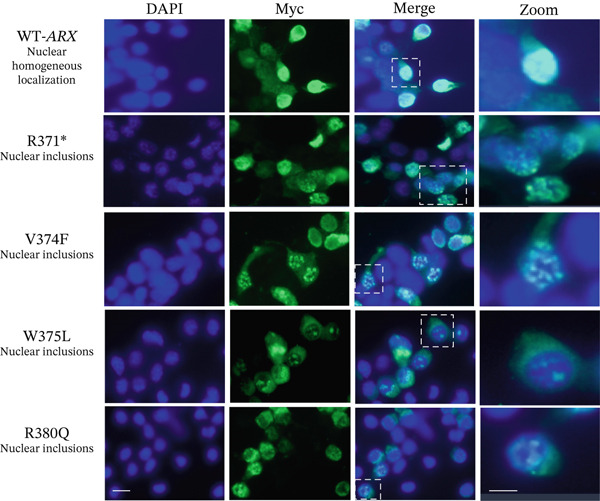
Atypical aggregated localization of *ARX* variants that showed activation of the reporter gene. N2a cells were transfected with plasmids encoding myc‐expressing *ARX* variants that were detected by immunofluorescence with anti‐myc antibody. N2a cell nuclei were stained with DAPI, and a magnified image of one cell is shown on the right, whereas WT‐*ARX* displayed a homogeneous nuclear staining, fluorescence microscopy revealed the presence of nuclear inclusions for all the variants that showed gain‐of‐function effects in luciferase experiments, suggesting abnormal localization and thus a possible dominant‐negative mechanism through possible sequestration of protein partners. Scale bar: 10 *μ*m.

**Figure 5:**
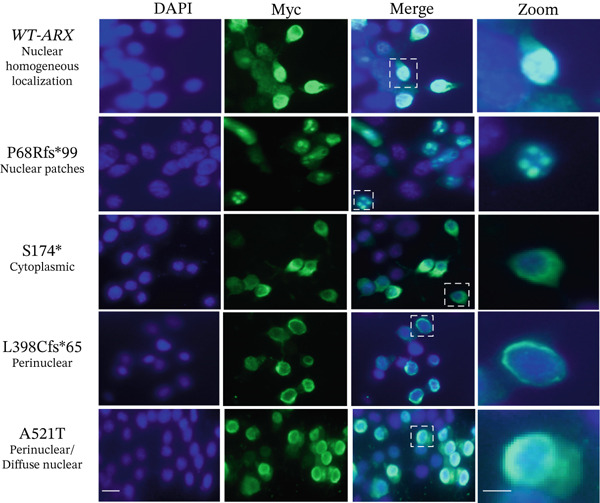
Abnormal patterns of subcellular localization of *ARX* variants. N2a cells were transfected with plasmids encoding myc‐expressing *ARX* variants that were detected by immunofluorescence with anti‐myc antibody. N2a cell nuclei were stained with DAPI, and a magnified image of one cell is shown on the right. Fluorescence microscopy revealed abnormal localization of certain variants, with cytoplasmic or perinuclear enrichment. Scale bar: 10 *μ*m.

To delve deeper into the potential dominant‐negative effects observed in specific *ARX* variants using the luciferase assay, we examined ARX protein localization using immunofluorescence microscopy. Proper nuclear localization of the *ARX* transcription factor is crucial for its interaction with DNA, and prior studies have demonstrated that variants in the nuclear localization signal (NLS) or homeodomain, such as p.R332P and p.P353R, disrupt *ARX* nuclear localization [33–35, 49]. Following transient transfection of myc‐tagged *ARX* variants into N2a cells, WT‐*ARX* protein predominantly exhibited a clear and homogeneous nuclear localization, as evidenced by the strong labeling confined to the nuclear regions stained with DAPI (Figure 4). Occasionally, faint cytoplasmic staining was observed in some cells, consistent with reports of ARX shuttling between the nucleus and cytoplasm [50]. In contrast, the variants p.R371∗, p.V374F, p.W375L, and p.R380Q showing an activation of the luciferase expression instead of repression (Figure 3b) all displayed an atypical non homogeneous nuclear localization, characterized by the formation of distinct nuclear inclusions, observed as intense localized, foci in transfected N2a cells (Figure 4). Of note, the variant p.L398Cfs∗65 showed a different pattern, with predominant perinuclear staining (Figure 5), again suggesting that the mechanism of physiopathogenicity may be different from the other variants in the homeodomain. Altogether, these findings indicate that the gain of transcriptional activity observed in the luciferase assay may be associated to the mislocalization or aggregation of mutant *ARX* protein in the nucleus. Such aggregates could potentially disrupt the localization or function of *ARX* interactors, consistent with a possible dominant‐negative mechanism.

Analysis of loss‐of‐function variants revealed variability in *ARX* localization. A few variants such as p.S174∗ exhibited abnormal cytoplasmic staining (Figure 5), whereas the majority, like p.L33P or p.Q163R, retained a nuclear distribution comparable with WT‐*ARX* (Figure S3). The p.P68Rfs∗99 mutation, which introduces a frameshift resulting in a truncated *ARX* protein with a novel C‐terminal extension of 99 arginine‐rich residues (Figure 2b), was particularly intriguing. Polyarginine regions are highly basic and can function as nucleic acid‐binding domains or mimic nuclear localization signals. However, in aberrant contexts, they may promote nonspecific interactions, phase separation, and the formation of toxic nuclear or cytoplasmic aggregates [51, 52]. This behavior may underlie the punctate nuclear patches observed for the p.P68Rfs∗99 variant (Figure 5).

Similarly, the variant p.L398Cfs∗65 has an atypical C‐terminal extension consisting of 65 proline‐rich residues (Figure 2b). Mechanistically, the polyproline tract likely adopts a left‐handed polyproline II helix, which enhances solubility and disrupts *β*‐sheet interfaces that are critical for tight nuclear aggregation [53, 54]. Proline‐rich sequences also serve as canonical docking sites for SH3 and WW domain‐containing proteins involved in nuclear transport and scaffolding. In cellular models, p.L398Cfs∗65 displayed a perinuclear ring‐like distribution (Figure 5) rather than forming chromatin‐associated inclusions, as observed with other dominant‐negative variants. This suggests that recruitment of specific factors to the polyproline extension may contribute to the variant′s mislocalization.

Interestingly, although the p.A521T variant did not show any significant effect on transcriptional activity, it showed in the majority of transfected cells a different type of staining combining perinuclear enrichment as well as a homogenous nuclear localization (Figure 5).

Taken together, these differences in protein localization suggest that certain mutations may affect *ARX* nuclear import or cause cytoplasmic retention, whereas others may impair its transcriptional activity despite proper apparent nuclear localization. Remarkably similar localization patterns were observed in 293T cells (data not shown), underscoring the cell‐type independence of these effects. Importantly, none of the observed nuclear inclusions stained positively with the Proteostat kit (data not shown) which specifically stains amyloid aggregates, suggesting that these inclusions or “aggregates” are not amyloid‐based.

### 3.4. Ability of Mutated Proteins to Interact With TLE1 and CtBP1 Corepressors

*ARX* has previously been shown to interact with corepressors TLE (Groucho/transducin‐like enhancer of split), in particular TLE1, via its OP (aa 27–34) and CtBP1 (C‐terminal‐binding protein 1) through its amino acids 512–562 [16, 31, 36] (Figure 6a). To further investigate the functional consequences of the studied *ARX* variants, we tested whether the overexpression of TLE1 or CtBP1 had any effect on the transcriptional activity of *ARX* variants. As shown in Figure 6, when transfected in N2a cells, TLE1, or CtBP1 had on their own a repressor activity on the luciferase reporter gene of approximately 25% (Figures 6b, 6c, and 6d). As none of these two proteins can bind DNA by themselves, they likely interact with other transcription factors endogenously expressed in N2a cells. When WT‐*ARX* was cotransfected with TLE1 or CtBP1, there was an additive effect of *ARX* and the corepressor, with a stronger repression activity on *Lmo1* promoter (reaching ~75%) (Figures 6b, 6c, and 6d), indicating a functional interaction between *ARX* and these corepressors that enhances transcriptional repression. Using this read‐out, we assessed whether *ARX* variants retained the ability to cooperate with TLE1 or CtBP1. As expected, variants that previously demonstrated aberrant activation instead of repression (Figure 3b), again displayed partial dominant‐negative effects, probably through sequestration, as they did not reach the level of TLE1 (Figure 6b) or CtBP (Figure 6c) alone. However, all these *ARX* variants were still able to interact with TLE1 and CtBP1 as additive repression was still observed when these variants were coexpressed with TLE1 or CtBP1 (Figure 6b,c). In contrast and as expected, truncated proteins, that lack the capacity to bind DNA and showed a loss‐of‐function effect in previous experiments (Figure 3a), did not have any additive effect with TLE1 (Figure 6d) but did not prevent TLE1 activity on its own. Similarly, these truncated proteins, lacking their domain of interaction with CtBP1, did not interfere with CtBP1 repression effect by itself (Figure S4a). As shown in Figures 6e and S4b, all missense variants were still able to interact with TLE1 or CtBP1 since an additive effect was observed for each variant, except p.L33P. Indeed, this mutation in the OP signal, which was used as a control in this experiment, is known to have lost its ability to interact with TLE1 [36].

Figure 6Transcriptional repression capacity of *ARX* variants in the presence of corepressors TLE1 or CtBP1. Plasmids encoding WT or mutant *ARX* protein were transfected in N2a cells along with either TLE1 or CtBP1, and their capacity to corepress transcriptional activity of the luciferase reporter gene under the control of *Lmo1* promoter was assessed. (a) Domains of ARX protein involved in the interaction with TLE1 (aa 27–34) and CtBP1 (aa 512–562) are shown. (b) Missense and nonsense mutations affecting the homeodomain were still able to interact with TLE1 as shown by the increased repression activity observed when TLE1 was cotransfected. However, the dominant‐negative effect of these variants was apparent as most of the cotransfection conditions did not reach the level of repression of TLE1 on its own. (c) Similar results were obtained with the same variants cotransfected with CtBP1. Note that p.L398Cfs∗65 and p.Ser450∗ were not tested as they lack the amino acids involved in CtBP1 interaction with *ARX*. (d) As expected, truncation variants that do not contain the homeodomain did not have any additive effect with TLE1. (e) All loss‐of‐function missense mutations kept their ability to interact with TLE1, except p.L33P, as shown by the additive effect of each *ARX* variant with TLE1, although they did not reach the level of translational repression of TLE1 + WT‐*ARX*. Comparison of each condition with TLE1 (b, d) or with CtBP1 (c), one‐way ANOVA with Dunnett′s post hoc test. ∗, *p* < 0.05; ∗∗, *p* < 0.01; ∗∗∗, *p* < 0.001, and ∗∗∗∗, *p* < 0.0001; ns: not significant. Comparison of the repression activity of each variant alone or in the presence of the corepressor (b, c, and e), Student′s *t*‐test: #, *p* < 0.05; ##, *p* < 0.01; ###, *p* < 0.001, and ####, *p* < 0.0001; ns: not significant.

**Figure:**
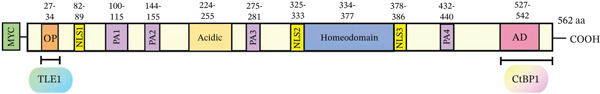
(a)

**Figure:**
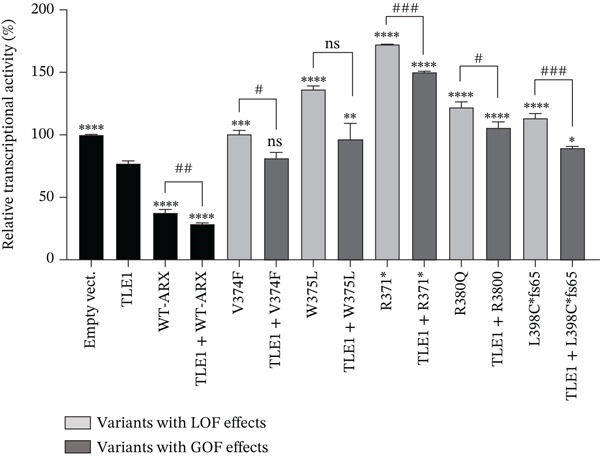
(b)

**Figure:**
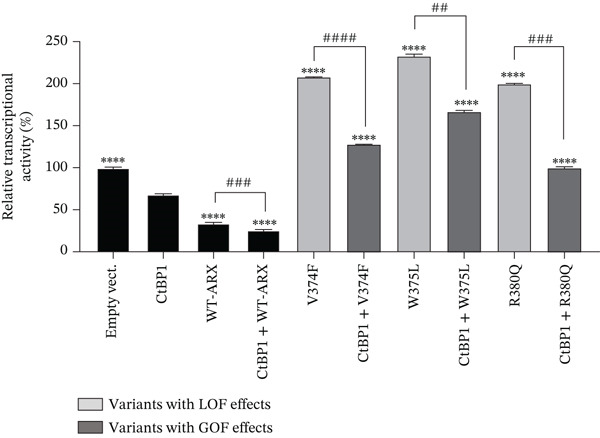
(c)

**Figure:**
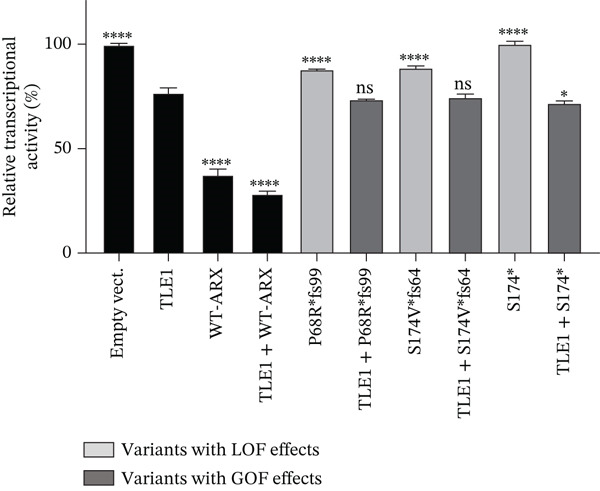
(d)

**Figure:**
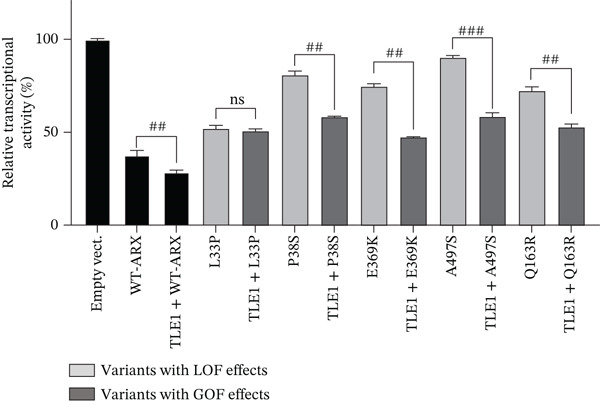
(e)

In conclusion, all tested variants, with the exception of truncated variants, were still able to exhibit an additive repressive effect with both corepressors, which further confirms that their ability to interact with TLE1 and CtBP1 is preserved. However, and most importantly, some of the mutants in the homeodomain may have possible dominant‐negative effects if mislocalized or unable to interact with DNA as they may prevent the accessibility of corepressors or proteins of the translational machinery to DNA.

### 3.5. *ARX* Variants Alter the Expression of Genes Involved in Brain Development

Since the binding of a transcription factor to a gene′s promoter does not necessarily imply transcriptional regulation, we next used RT‐qPCR to evaluate whether *ARX* variants differentially regulate the expression of the endogenous *Lmo1* gene that was assessed in luciferase assays, along with two other target genes of *ARX*, *Olfm1*, a gene with abundant expression in the brain contributing to the regulation of axonal growth in the embryonic and adult central nervous system, and *L1 cell adhesion molecule* (*L1cam*), a cell adhesion molecule crucial for neuronal migration, axonal guidance, and synapse formation. *Olfm1* and *L1cam* were previously identified by our group as targets of *ARX* [42]. Both genes were found enriched in *ARX* ChIP (chromatin‐immunoprecipitated) samples from *ARX*‐transfected N2a cells and their expression was clearly down‐regulated in *ARX*‐transfected cells [42].

As shown in Figure 7, none of the tested variants, including p.A521T, which presented a repressive activity similar to WT‐ARX in the luciferase assay (Figure 3a, bottom panel), was able to efficiently repress the expression of any of the three endogenous *ARX* direct targets. These findings underscore the sensitivity of RT‐qPCR in detecting changes in gene expression, revealing that all tested *ARX* variants induced significant alterations in the normal expression patterns of target genes.

Figure 7Effect of *ARX* variants on the endogenous expression of three of its direct targets in N2a cells. N2a cells were transfected with plasmids encoding myc‐tagged WT or ARX variants, and 24 h later, RNA was isolated and reverse transcribed into cDNA. The expression of *ARX* target genes, *Lmo1* (a), *L1cam* (b), and *Olfm1* (c) was tested. Comparison of each condition with WT‐*ARX*, one‐way ANOVA with Dunnett′s post hoc test. ∗, *p* < 0.05; ∗∗, *p* < 0.01; ∗∗∗, *p* < 0.001; and ∗∗∗∗, *p* < 0.0001; ns: not significant.

**Figure:**
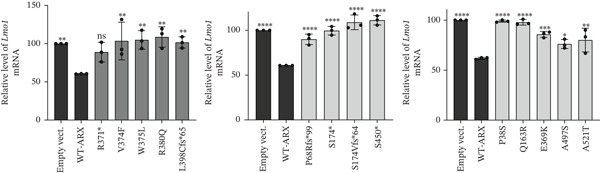
(a)

**Figure:**
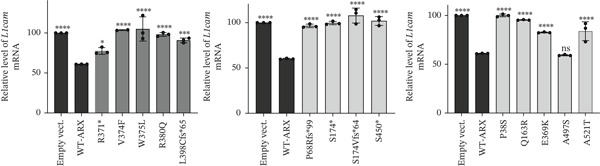
(b)

**Figure:**
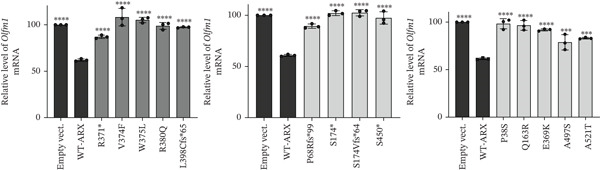
(c)

## 4. Discussion

Since the first identification of *ARX* as a gene involved in a broad spectrum of NDDs in 2002, numerous mutations have been described. However, the majority of variants have been identified in males due to the historical lack of routine genetic screening in females for X‐linked related disorders, particularly in isolated cases. It is only in recent years, with the more widespread use of next‐generation sequencing, that reports of *ARX* mutations in female patients have increasingly emerged [29, 55–59]. We here present a set of simple tests that were developed to investigate the functional consequences of several *ARX* variants. Data obtained are summarized in Table 2. These tests are sufficiently sensitive to reveal that variants outside canonical functional domains can disrupt ARX function, which emphasizes the need for experimental validation beyond in silico prediction.

**Table 2:** Summary of the data obtained for each variant.

Variants studied	Effects on transcriptional repression	Still contains the homeodomain	Subcellular localization	Interaction with Tle1 or Ctbp1 likely preserved?	Repression of target genes
p.L33P	Loss‐of‐function	Yes	Normal	Not with Tle1	Decreased repression
p.P38S	Loss‐of‐function	Yes	Normal	Yes	Decreased repression
p.P68Rfs∗99	Loss‐of‐function	No	Nuclear patches	Not with CtBP1	Decreased repression
p.Q163R	Loss‐of‐function	Yes	Normal	Yes	Decreased repression
p.S174Vfs∗64	Loss‐of‐function	No	Only nuclear	Not with CtBP1	Decreased repression
p.S174∗	Loss‐of‐function	No	Only cytoplasmic	Not with CtBP1	Decreased repression
p.P353R	Complete loss‐of‐function	Yes but likely not functional	Predominantly cytoplasmic	Yes	Decreased repression
p.E369K	Loss‐of‐function	Yes but likely not functional	Normal	Yes	Decreased repression
p.R371∗	Dominant‐negative	Only part of it	Nuclear inclusions	Not with CtBP1	Decreased repression
p. V374F	Dominant‐negative	Yes but likely not functional	Nuclear inclusions	Yes	Decreased repression
p.W375L	Dominant‐negative	Yes but likely not functional	Nuclear inclusions	Yes	Decreased repression
p.R380Q	Dominant‐negative	Yes but likely not functional	Nuclear inclusions	Yes	Decreased repression
p.L398Cfs∗65	Dominant‐negative	Yes	Perinuclear inclusions	Not with CtBP1	Decreased repression
p.S450∗	Loss‐of‐function	Yes	Normal	Not with CtBP1	Decreased repression
p.A497S	Loss‐of‐function	Yes	Normal	Yes	Decreased repression
p.A521T	Normal repression	Yes	Perinuclear	Yes	Decreased repression

It is important to note that PA expansions have not been included in this study. These expansions have been extensively characterized and discussed in previous reports [20, 22, 26, 32, 34, 60–63].

**Figure 8:**
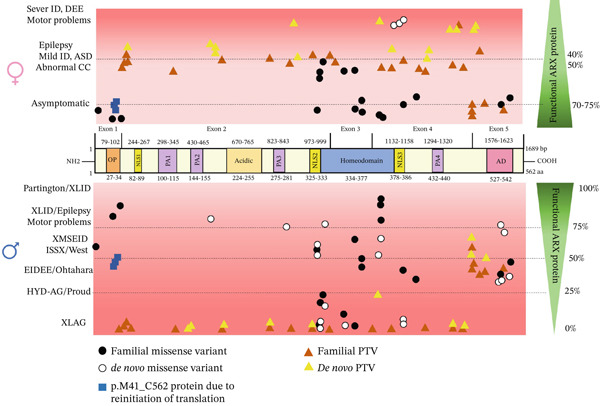
Localization of *ARX* mutations in female and in male individuals reported in the literature. Mutations reported in males (below, in blue) and females (above, in black) are associated with their corresponding phenotypes, with an estimation (based on available functional data or phenotype severity in males) of the residual function of the mutant protein shown on the right side of the figure. Although there is a clear correlation in males between the localisation and the nature of the mutations and the severity of the phenotype, it is less clear in females, showing that other factors, such as X‐chromosome inactivation, NMD, possibly dominant‐negative effects, and environmental factors may influence the severity of the phenotype.

By integrating information from the existing literature with some of our functional data, we next present an attempt to model genotype/phenotype correlations in both male and female individuals with an *ARX* variant and to offer a general overview of current knowledge on the subject (Figure 8; Table S1), with the hope that this may assist clinicians in predicting the phenotype severity, particularly in the case of prenatal genetic diagnosis. This proposed model takes into account the estimated residual function of the protein that is evaluated both on available published functional data and on the severity of the phenotypes observed in males. Additional data, such as the clinical description of the patients and X‐inactivation status, when available, are detailed in Table S1. It should be noted that this estimated residual function of the protein is, for some variants, hypothetical and may be confirmed or refuted as new descriptions of patients and variants become available.

### 4.1. Variants Resulting in the Absence of *ARX* Protein

A major conclusion from the analysis of Table S1 is that, in both sexes, 50% residual functional *ARX* protein is generally insufficient for physiological cellular development and cerebral functions. As shown in Figure 8 and Table S1, with the exception of missense mutations in the homeodomain, mutations associated with clinical findings in females are PTVs, which likely trigger NMD, a cellular quality control process that degrades mutant mRNA transcripts before translation (see for review [64]).

According to the rules of NMD, PTV located near the 3 ^′^ end of genes, specifically in the final coding exon or in the last 50–55 nucleotides of the penultimate coding exon, are expected to escape NMD [65]. In such cases, rather than being degraded, the mRNA is translated into a truncated protein. In the case of *ARX*, this NMD rule probably applies as males with PTVs in the last exon do not have the most extreme XLAG phenotype (Figure 8). In addition, it has been suggested that premature large exons (greater than 400 nucleotides, which is the case of exon 2 of *ARX*) may have increased rates of NMD escape [66]. However, this seems unlikely for *ARX* when considering the male phenotype associated with PTVs in exon 2 (Figure 8). In any case, if some PTVs do escape NMD, our results with p.P68Rfs∗99, p.S174∗, and p.S174Vfs∗64 indicate that the produced truncated proteins result in a complete loss‐of‐function regarding transcriptional activity, which is consistent with the loss of their homeobox DNA‐binding domain.

For the males, all cells are supposed to express a similar amount of functional *ARX* protein, explaining why the genotype/phenotype relationship is clearer. The variable phenotype in heterozygous women has been attributed to X‐chromosome inactivation. Indeed, in females, X inactivation adds a level of complexity, as approximately half of the cells express a WT *ARX* protein and the other 50% of cells express a mutant protein with or without residual function. However, in cases of skewed X inactivation (defined as a ratio of 80:20) or if 60%–70% of brain cells express the WT allele, this may make a difference and result in a more or less severe phenotype than expected. Unfortunately, the X inactivation status is not always available and when it is, it has often been tested in blood cells, which may give a different pattern than that seen in the brain. Although some of the published results of X inactivation may confirm its impact on phenotype severity (Table S1), it is difficult to be entirely conclusive, given the small number of female cases reported, the possible difference between tissues (blood vs. brain) and the possibility of crossing overs between the androgen receptor (AR) and *ARX* loci.

Furthermore, the situation is further complicated by the fact that *ARX* is not like *DCX* (*doublecortin*), another X‐linked gene that encodes a microtubule‐binding protein and therefore has primarily a cell‐autonomous function in neuronal migration. Neurons expressing WT *DCX* migrate correctly to the cortical plate in the developing cerebral cortex whereas neurons expressing the mutated form exhibit migratory defects and remain in subcortical regions, leading to a cerebral malformation called subcortical laminar heterotopia (or “double cortex”). For *ARX*, the situation is more complex as both cell‐autonomous and noncell autonomous roles of this protein have been described [13, 67, 68], meaning that cells expressing mutated *ARX* may, at least partially, benefit from cells expressing the WT gene, for example through the secretion of signaling proteins involved in brain development. All these reasons probably explain the significant phenotypic variability observed in females who have an estimated residual ARX function of 50% (Table S1 and Figure 8).

### 4.2. Variants Affecting the mRNA or Protein Stability, Thus Reducing the Total Amount of *ARX* Protein Produced

Considering mutations in the N‐terminal region of the protein, the p.S2C mutation, which has been described in several members of the same family, is thought to cause abnormal folding and possible aggregation of the resulting protein, leading to its degradation and thus reducing the total amount of *ARX* protein available [69]. Accordingly, the phenotype of affected males of this family was described as closely resembling Partington syndrome, a condition typically associated with PA expansions in the second PA tract of ARX, and characterized by ID, hand dystonia, and dysarthria. Additional neurological features, including epilepsy and EEG abnormalities, were observed in some patients, suggesting variable expressivity. In contrast, female carriers were asymptomatic (Table S1), which is consistent with the fact that the mutant protein likely has some residual function (estimated at approximately 75%–85%). The important phenotypic variability observed among males of the family has been described as potentially attributable to the existence of other environmental or genetic determinants. Most importantly, the phenotype also likely depends on the interindividual capacity to deal with misfolded *ARX* and the associated cellular machinery involved in inducing defense systems aimed at decreasing the mutant *ARX* protein load. This includes the expression of heat‐shock proteins, induction of the unfolded protein response (UPR), and induction of autophagy or mitophagy, similar to what is observed with PA expansions [32, 70], or in neurodegenerative diseases linked to protein misfolding such as Parkinson′s or Alzheimer′s diseases.

Similarly, three different nonsense mutations targeting the first 28 amino acids of *ARX* have been described, leading to an unexpectedly milder phenotype than XLAG. Two studies have shown that for p.E12∗ and p.Y27∗, there is translation reinitiation at the methionine 41, resulting in the production of a reduced amount of a mutant protein lacking the OP but keeping some residual function [44, 45]. Of note, our results confirm these authors′ observations that nonsense mutations or PTV located downstream of methionine 41 do not lead to translation reinitiation (Figure 2 and Figure S1). It has been previously shown that the N‐terminal part of the protein, which is lacking here, acts as a mild transcriptional repressor domain, probably through its OP [16, 36]. This suggests that the reduced protein levels and the partial loss of the repressive transcriptional activity of *ARX* are sufficient to disrupt the normal development of GABAergic interneurons in males, leading to severe epileptic encephalopathies such as IESS (Infantile Epileptic Spasm Syndrome) or EIDEE (Early Infantile Developmental and Epileptic Encephalopathy), while allowing heterozygous females to remain asymptomatic with normal cerebral development due to the residual function of the resulting p.M41_C562 protein in approximately half of their cells.

### 4.3. Impact of Missense and Nonsense Mutations Located in the *ARX* Homeodomain

Concerning missense mutations located in the *ARX* homeodomain, their functional impact has been extensively characterized by Shoubridge et al. Most of them have been shown to have a loss‐of‐function effect due to either decreased DNA target recognition or binding, or altered nuclear localization due to impaired interaction with importins [32, 33, 35, 49]. The severity of the phenotypes in males appears to be directly correlated to the degree of the molecular impairment. Mutations in key residues of the homeodomain lead to XLAG whereas variants in less critical residues such as p.R330 or p.R358 result in slightly less severe phenotypes. Here, we describe a novel variant in the homeodomain affecting the first residue E369 of helix 3 of *ARX* homeodomain. Although this residue is not predicted to be one of the most important for DNA binding specificity or affinity, it is nevertheless predicted to be in contact with DNA. It is thus not surprising that we observed decreased transcriptional repression, even if this mutation does not change the subcellular localization of the protein.

Another key finding coming from this study is the description of dominant‐negative effects associated *with three de novo variants* recently described in women (p.V374F, p.W375L, and p.R380Q). Interestingly, all three variants are located in helix 3 of the homeodomain, a critical domain responsible for DNA target recognition. When expressed in N2a or 293T cells, these variants caused abnormal nuclear inclusions, which are potentially indicative of protein misfolding that may disrupt normal DNA recognition. Indeed, helix 3 of the homeodomain plays a central role in DNA binding, and recent evidence emphasizes the importance of specific residues within this domain. According to a recent study, the valine residue in Position 374 of *ARX* homeodomain is important for DNA recognition specificity, whereas the tryptophan in Position 375 is important for both DNA affinity and specificity [71]. What remains unclear is whether the dominant‐negative effects observed in this study are also present in vivo or if they result from the strong expression level of these variants in our experimental assays.

Interestingly, we also found a similar dominant‐negative effect with the truncated proteins resulting from p.R371∗ (this study) and p.W375∗ (unpublished data) variants. The latter variant was identified in a young girl with primary immunodeficiency, severe intractable diarrhea, pneumonia, and dysmorphic features. Unfortunately, no neurological or developmental information about this patient was available and she has been lost to follow‐up. Both of these PTVs are expected to be subjected to NMD. However, the severity of the resulting phenotype could still be influenced by a partial efficiency of NMD and the effects of X‐chromosome inactivation. Indeed, NMD has been shown to be only partially efficient [72] and several examples of PTV leading to more severe phenotypes when the mRNA encodes a partially functional protein but with different properties have been uncovered. Accordingly, these different variants are de novo and have been described to result in particularly severe neurological phenotypes in women, which could be in agreement with a dominant‐negative effect in vivo. However, it remains challenging to draw definitive conclusions at this stage due to the limited number of patients reported and the difficulty in obtaining relevant tissues for studies. In any case, for genetic counseling purposes, it is important to consider that missense variants and PTV in the homeodomain can lead to very severe phenotypes, in both females and males.

### 4.4. Variants in the Two Last Exons of *ARX*

According to the rules of NMD, PTVs should escape NMD if they occur approximately 50–55 nucleotides before the exon 4/5 junction, corresponding to amino acid Position 465 in *ARX* protein. However, clinical observations suggest that PTVs resulting in premature truncation up to amino acid 476 cause XLAG in males (Figure 8). This implies that either the resulting truncated protein is unstable and rapidly degraded, or that it lacks an essential functional domain critical for neuronal development. Consistent with these clinical and molecular findings, earlier in vitro studies that dissected the *ARX* protein into functional regions mapped an essential repressor domain approximately between aa 400–495 [16, 37]. Loss of this repressor domain likely results in significantly reduced transcriptional repressor activity of *ARX*, which in turn disrupts normal GABAergic interneuron development, underlying severe epileptic phenotypes. The severe defect in transcription repression we observed with the p.Ser450∗ truncated protein also supports this hypothesis.

Among the genes repressed (directly or indirectly) by *ARX* is *L1CAM*, which encodes a cell adhesion molecule involved in neuronal migration and differentiation and which has been associated with CC agenesis [73]. It is possible that *ARX* and *L1CAM* act within the same transcriptional pathway, thereby contributing to common neurodevelopmental phenotypes. Further research will be needed to elucidate the precise role of *L1CAM* in *ARX*‐related phenotypes and confirm its functional interaction with *ARX*.

PTV removing the AD tend to result in milder phenotypes in females but are associated with EIDEE in males. Interestingly, patients with mutations in this transcriptional activator domain also have severe motor defects, including spastic quadriplegia and are in some cases wrongly diagnosed as “cerebral palsy.” We found that ARX positively regulates the development of striatal medium spiny neurons (manuscript in preparation), which agrees with the hypothesis that a loss of *ARX*′s activating transcriptional activity may impair basal ganglia development, potentially contributing to the severe motor phenotypes observed in these patients.

### 4.5. Impact of Nonconservative Missense Variants in *ARX*

The interpretation of missense variants identified in exon 2 and outside main functional domains of the protein remains particularly challenging in the absence of functional testing. Among them, the variants p.R264Q and p.A279T have, to date, only been described in female carriers, and are associated with relatively mild phenotypes. The two other variants, p.Q163R and p.G286S, although not located within known functional domains of the ARX protein, exhibit a partial loss‐of‐function effect in our tests (Figure 3a for p.Q163R and data not shown for p.G286S) without significantly affecting the nuclear localisation of the protein (Figure S3 and data not shown). This suggests that the observed loss of repression could be due to changes in protein conformation or altered interactions with cofactors, rather than incorrect subcellular localization of the protein.

The functional consequences of variants p.A497S and p.A521T, both located outside canonical functional domains, are even more difficult to predict in the absence of comprehensive experimental analyses. The variant p.A521T has previously been reported in male patients with ID but is also found in the general population. Depending on the prediction software, this variant gives contradictorily results concerning its pathogenic or benign impact (https://mobidetails.chu-montpellier.fr/api/variant/706130/browser/). Accordingly, this variant did not significantly alter transcriptional repression in luciferase assays, suggesting that basic DNA‐binding and repression activity of *ARX* remain intact. However, RT‐qPCR experiments in transfected cells indicated a significant decrease in the repression of endogenous *ARX* targets, pointing toward a possible defect in protein‐protein interactions or chromatin recruitment dependent on the cellular context. Similarly, the variant p.A497S was identified in a young boy who died in infancy. The pathophysiologic role of this variant is difficult to assess, as it was present in only 12% of sequencing reads in the heart and 7% in the brain. Nevertheless, its identification raises a compelling hypothesis in light of a case described by [56] in which a maternal uncle of the proband reportedly died at 1 month of age from sudden infant death syndrome. Although definitive causality cannot be established, the recurrence of early infant death in association with *ARX* variants warrants further investigation, particularly in light of the gene′s roles in heart and neuronal function.

## 5. Conclusion

To summarize, the data presented in this study reinforce the concept that the impact of *ARX* mutations operates through a mechanism of haploinsufficiency, in which the quantity and functionality of the protein are essential for proper neurodevelopment. In both males and females, reduced gene dosage, whether due to NMD, impaired protein stability, or functional inactivation, can lead to a broad spectrum of NDDs. Importantly, the severity and nature of these disorders are influenced by the position and type of the variant, particularly with regard to the conservation of the homeodomain, the N‐terminal and central transcriptional repressor domains (amino acids 1–66 and 400–495), and the C‐terminal transactivation domain (amino acids 527–542). Variants that preserve protein expression but disrupt transcriptional repression or interaction with cofactors can still have deleterious effects, as demonstrated by luciferase reporter and RT‐qPCR assays. In females, predicting phenotype severity remains particularly challenging, as it depends not only on the molecular features of the variant but also on factors such as X‐inactivation patterns, NMD efficiency, genetic background, and environmental modifiers. This highlights the complex interplay between genetic and epigenetic regulation in shaping disease expression in heterozygous carriers.

In conclusion, this study significantly advances our understanding of the molecular mechanisms underlying *ARX*‐related disorders, offering a refined framework for interpreting novel variants. Through the functional characterization of both known and newly identified missense mutations, we demonstrate that even variants outside canonical domains can profoundly alter *ARX* function. These insights are essential for improving diagnostic precision, guiding genetic counseling, and ultimately guiding the development of targeted therapeutic strategies aimed at restoring *ARX* function or compensating for its loss. Continued research into *ARX* regulation, variant‐specific consequences, and downstream molecular pathways will be pivotal in translating these molecular findings into clinical applications.

## Author Contributions

Conceptualization: R.F., C.V., and G.F.; data curation: investigation and writing—review and editing: R.F., A.F., A.C.E.H., P.C., J.M., A.S., S.R., A.D., M.G., A.C., C.V., and G.F.; supervision: C.V. and G.F.; writing—original draft: R.F., C.V., and G.F.

## Funding

This study was supported by the Fondation Jérôme Lejeune, the Association Gaëtan Saleun, the Université de Bretagne Occidentale (UBO), the IBSAM, and the French National Institute of Health and Medical Research (INSERM). Rasha Faraj was supported by a PhD grant from Brest Métropole and from the Ministère de l′Enseignement Supérieur et de la Recherche.

## Ethics Statement

Clinical information, laboratory findings, imaging, neuropathological data, and genetic testing for the individuals listed in Table 1 were collected and evaluated as part of standard clinical care. Genetic results were shared upon written informed consent obtained from all individuals or their parents

## Conflicts of Interest

The authors declare no conflicts of interest.

## Supporting Information

Additional supporting information can be found online in the Supporting Information section.

## Supporting information


**Supporting Information 1** Description of the patients (Patients 8 and 15 from Table 1).

**Supporting Information 2** Table S1 lists *ARX* mutations reported in the literature and the residual function estimated from functional data when available. Male subjects are indicated in black, female subjects are indicated in blue. Table S2 lists the primers used in this study.

**Supporting Information 3** Figure S1 presents Western‐blot analysis showing a faint band (indicated by an arrow) corresponding to a longer peptide likely produced by RNA editing of the variant or stop codon readthrough of the variant p.S174V∗fs64. The blot was detected using a polyclonal antibody directed against *ARX* homeodomain. Figure S2 presents Transcriptional activation capacity of the different *ARX* variants in 293T cells. Plasmids encoding WT‐*ARX* or mutant constructs were transfected in 293T cells, and their capacity to activate the expression of the luciferase reporter gene under the control of *Lmo1* promoter was assessed. (a) Several truncation mutations as well as missense variants located outside *ARX* homeodomain caused severe loss‐of‐function (represented in light gray). (b) In contrast, missense or truncation variants located within or in close proximity but downstream *ARX* homeodomain caused an apparent gain‐of‐function (represented in dark gray) with an overactivation of the expression of the reporter gene under the control of *Lmo1* promoter region. (a–b) Comparison of each condition with WT‐*ARX*, one‐way ANOVA with Dunnett′s post hoc test. ∗, *p* < 0.05; ∗∗, *p* < 0.01; ∗∗∗, *p* < 0.001; and ∗∗∗∗, *p* < 0.0001. Truncation variants are represented in dark gray whereas missense variants are in light gray. (c) Schematic representation of the variants that showed an apparent gain‐of‐function in luciferase experiments. Figure S3 presents several variants exhibit similar immunolabeling patterns to WT‐*ARX* in N2a Cells. N2a cells were transfected with plasmids encoding myc‐expressing variants, that were detected by immunofluorescence with anti‐myc antibody. N2a cell nuclei were stained with DAPI. Several variants had similar pattern of subcellular expression as WT‐ARX with a majority of diffuse nuclear staining and occasional faint cytoplasmic labeling. Scale bar: 10 *μ*m. Figure S4 presents Transcriptional repression capacity of the different *ARX* variants in the presence of the corepressor CtBP1. Plasmids encoding WT or *ARX* mutant constructs were transfected in N2a cells along with CtBP1, and their capacity to repress transcriptional activity of the luciferase reporter gene under the control of *Lmo1* promoter was assessed. (a) As expected, truncated proteins lacking the homeodomain and the C‐terminal part of the protein responsible for the interaction with CtBP1 did not have any effect on CtBP1 capacity to repress transcription by itself. Comparison of each condition with CtBP1, one‐way ANOVA with Dunnett′s post hoc test. ∗∗∗∗, *p* < 0.0001; ns: not significant. (b) Proteins with missense mutations located in the C‐terminal part of *ARX* were still able to interact with CtBP1 and had additive effects, showing that these residues are probably not involved in this interaction. Comparison of the repression activity of each variant alone or in the presence of the corepressor CtBP1, Student′s *t*‐test: ##, *p <0.01*; ###, *p <0.001*; and ####, *p <0.0001*.

## Data Availability

All data will be made available on request.
