# Supplementary material for: Cellular Functional Analyses of ARX Variants Reveal New Insights Into Genotype–Phenotype Correlations in Neurodevelopmental Disorders Among Male and Female Patients
Source: Hum Mutat. 2026 Apr 7;2026:4732622. doi: 10.1155/humu/4732622 (PMC13058441; doi:10.1155/humu/4732622)
Supplement: Supplementary file 1 — Supporting Information 1 Description of the patients (Patients 8 and 15 from Table 1). [file HUMU-2026-4732622-s002.docx]

**Cellular Functional Analyses of *ARX* Variants Reveal New Insights into Genotype-Phenotype Correlations in Neurodevelopmental Disorders among Male and Female Patients**

Rasha Faraj^1,2^, Audrey Farrugia^3-4^, Anna C.E. Hurst^5^, Pierre Conan^2^, Jennifer Martin^2^, Audrey Schalk^6^, Sylvia Redon^7-8^, Aline Dubos^4^, Mathilde Gras^9^, Aurore Curie^10-12^, Cécile Voisset^1,2^, Gaëlle Friocourt^1,2,8^

**Description of the patients**

Patient 8 of Table 1 is a male who first presented to genetics clinic at 8 months of age for evaluation of microcephaly and seizures. He was born at 38 weeks’ gestation to a 31 years-old G1 mother via vaginal delivery with no prenatal or neonatal complications. Birth weight was 2.97 kg (in the 25-50^th^ percentile), length 48.25 cm (in the 25-50^th^ percentile), and head circumference 30.5 cm (below the 50^th^ percentile for a 33-week gestation infant). The initial small head circumference was attributed to molding during delivery, but at initial paediatrician visit, an MRI was scheduled for 1 month of age. This showed absent corpus callosum, subtle loss of white matter volume in the left cerebral hemisphere, and small focus of subacute subdural haemorrhage overlying the right cerebellar hemisphere with additional fluid collections over the right and left cerebellar hemisphere that may have been due to prior subdural or epidural haemorrhage.

He developed seizures and first sought medical attention between 6-8 weeks of life for these events described by neurology as infantile spasms with an emerging pattern of tonic seizures concerning for Lennox-Gastaut pattern.

Medically, he had laryngomalacia and a supraglottoplasty was performed for stridor. Polysomnography showed episodes of apnea and he was started on 0.5 L nasal cannula support. A nasogastric tube was placed for medication administration; later G-tube was placed. Ophthalmology diagnosed him with cortical vision impairment.

Developmentally, at 8 months of age he had emerging, limited head control, rolls front-to-back and was attempting to sit with assistance.

At the 8-month evaluation, his height was 68 cm (in the 8^th^ percentile), weight 9.75 kg (in the 85 percentile), and head circumference 38.5 cm (far below the first percentile, Z-score -4.96). Physical examination was significant for stridor, profound microcephaly and bitemporal narrowing, epicanthal folds, long palpebral fissures that gives appearance of slightly shortened inner canthal distance (although IC is in the 50^th^ percentile), short columella, long philtrum, tented upper lip with upturned vermillion border.

A prior microarray was negative. An epilepsy panel sent to a commercial laboratory demonstrated ARX c.1105G>A, p.Glu369Lys variant of uncertain significance, but in order to rule out other contributing factors, clinical whole genome sequencing was performed. This showed the variant was not maternally inherited. The genome results did show carrier status for a paternally inherited *ADAR* c.577C>G, p.Pro193Ala pathogenic variant (carrier for Aicardi-Gouitieres type 6), and no second variant was identified.

He is now 5 years old and has persistent intractable multifocal epilepsy in the form of Lennox-Gastaut syndrome, with an average of 5 seizures a week. EEGs have demonstrated multifocal spike discharged. His medication regimen includes fenfluramine, cannabidol, and a ketogenic diet.

Patient 15 (Table 1) is a male infant born at 33 weeks of amenorrhea, who died two months after birth during the night while sleeping in his mother's arms on a sofa. He was the couple's only child. Microbiological, toxicological, and anatomopathological investigations did not identify a cause of death. As a result, the diagnosis of sudden infant death syndrome (SIDS) was established.

Genetic investigations (NGS with a panel of 181 genes targeting those involved in cardiopathies, channelopathies and epilepsy) identified a missense variant in the *ARX* gene (NM_139058.2:c.1489G>T, p.Ala497Ser). The variant was found in mosaic form, present in 12% of reads from DNA extracted from cardiac tissue, according to our panel analysis. We also performed whole-exome sequencing from a brain sample, where the mosaic variant was detected at 7%. The nucleotide is highly conserved (phyloP: 4.81 [-14.1;6.4]) up to the zebrafish (across 10 species). Prediction tools are consistent and suggest the pathogenicity of the variant:

- **SIFT (v6.2.0)**: Deleterious (score: 0, median: 3.73)
- **MutationTaster (v2013)**: Disease-causing (prob: 1)
- **PolyPhen2**: Probably damaging

In order to confirm the pathogenicity of this variant, a functional study was carried out.

All analytical experiments were conducted at the request of judicial authorities. Informed consent was not necessary. The identity of patient is completely concealed.

**Supplementary Table 1: list of *ARX* mutations reported in the literature and the residual function estimated from functional data when available.** Males are indicated in black, females are indicated in blue.

**Supplementary Table 2: List of primers used in this study**

| **Primer name** | **Primer sequence** |
| --- | --- |
| hTLE1-Xho-GFP-F | CCGGACTCAGATCTCGAGCGATGTTCCCGCAGAGCC |
| hTLE1-Xho-GFP-R | TCCCGGGCCCGCGGTACCTCAGTAGATGACTTCATAGACTG |
| hCtBP1-Hind-FLAG-F | GGATGACGATGACAAGCTTGAGATGTCAGGCGTCCGACC |
| hCtBP1-EcoRI-FLAG-R | GATCTATCGATGAATTCGGCTACAACTGGTCACTGG |
| mLmo1-F | TAAGCTAATGGCGGGCACCT |
| mLmo1-R | CTCGCTCTCACCAGAGTGCA |
| mCalb2-F | AGGGAGTTGAAAGGAGAGGAGGAA |
| mCalb2-R | GGGAAGAATTGTGGGAGGG |
| mCdh2-F | GCATGAATTGGTAACAGGGTCAGG |
| mCdh2-R | CCACAGCATTATCTAGCCCTTC |
| hARX-L33P-F | CTCCTCCGGCCCGGGATGCTGTCGA |
| hARX-L33P-R | TCGACAGCATCCCGGGCCGGAGGAG |
| hARX-P38S-F | CGCATTTTGCACGAGCTCCTCCGGCCC |
| hARX-P38S-R | GGGCCGGAGGAGCTCGTGCAAAATGCG |
| hARX-P68Rfs-F | CGCTGCTGCTCTTGAGCCTTGCACGG |
| hARX-P68Rfs-R | CCGTGCAAGGCTCAAGAGCAGCAGCG |
| hARX-S174V-F | CATCAGCCGCAGCAAAGTCGTACCGCGAG |
| hARX-S174V-R | CATCAGCCGCAGCAAAGTCGTACCGCGAG |
| hARX-S174*-F | CGTTCTCGCGGTACTACTTGCTGCGGCTG |
| hARX-S174*-R | CAGCCGCAGCAAGTAGTACCGCGAGAACG |
| hARX-P353R-F | TGGTGAAGACGTCCCGGTAGTGCGTCTTC |
| hARX-P353R-R | GAAGACGCACTACCGGGACGTCTTCACCA |
| hARX-E369K-F | TGGACTCGGGCCTTGGTCAAGTCCAGC |
| hARX-E369K-R | GCTGGACTTGACCAAGGCCCGAGTCCA |
| hARX-R371*-F | CAGACCTGGACTCAGGCCTCGGTCAAG |
| hARX-R371*-R | CTTGACCGAGGCCTGAGTCCAGGTCTG |
| hARX-V374F-F | GTTCTGGAACCAGAACTGGACTCGGGCCT |
| hARX-V374F-R | AGGCCCGAGTCCAGTTCTGGTTCCAGAAC |
| hARX-W375L-F | GACGGTTCTGGAACAAGACCTGGACTCGG |
| hARX-W375L-R | CCGAGTCCAGGTCTTGTTCCAGAACCGTC |
| hARX-R380Q-F | GCGCCACTTGGCCTGACGGTTCTGGAA |
| hARX-R380Q-R | TTCCAGAACCGTCAGGCCAAGTGGCGC |
| hARX-L398C-F | CGGGAAGGGCAGCCAGGGGGGTGG |
| hARX-L398C-R | CCACCCCCCTGGCTGCCCTTCCCG |
| hARX-S450*-F | AGGCTGGCCTAGCCCGGAGGCGG |
| hARX-S450*-R | CCGCCTCCGGGCTAGGCCAGCCT |
| hARX-A497S-F | TCTCAGGAGCGAGGCCGCGGTCG |
| hARX-A497S-R | CGACCGCGGCCTCGCTCCTGAGA |
| hARX-A521T-F | GCCGCCGTGGTCGGGTCGGCC |
| hARX-A521T-R | GGCCGACCCGACCACGGCGGC |
| mLmo1-qF | GTATCACCTTGACTGCTTCGCC |
| mLmo1-qR | CCTCATAGTCCACCTGGCACAA |
| mOlfm1-qF | TCAAGGTTCGGGTCCTGGATGA |
| mOlfm1-qR | GGTGAGAGGTAAAGTTGTCCGTG |
| mL1cam-qF | TGTGGAAGTGGAGGAAGGAGAA |
| mL1cam-qR | ATAGGTCTCCATTCTGGCCCAT |
| mAct-qF | AGAGGGAAATCGTGCGTGACA |
| mAct-qR | CACTGTGTTGGCATAGAGGTC |
