# Supplementary material for: Cellular Functional Analyses of ARX Variants Reveal New Insights Into Genotype–Phenotype Correlations in Neurodevelopmental Disorders Among Male and Female Patients
Source: Hum Mutat. 2026 Apr 7;2026:4732622. doi: 10.1155/humu/4732622 (PMC13058441; doi:10.1155/humu/4732622)
Supplement: Supplementary file 2 — Supporting Information 2 Table S1 lists ARX mutations reported in the literature and the residual function estimated from functional data when available. Male subjects are indicated in black, female subjects are indicated in blue. Table S2 lists the primers used in this study. [file HUMU-2026-4732622-s001.pdf]

Phenotype	Mutation (DNA) (NM_139058)	Mutation (protein)	Estimated residual protein function	Family	References
XLAG	exon1-2 del	no protein produced	0% normal function	LP94-058	Dobyns, Am J Med Genet, 1999 (case 1), Kitamura, Nat Genet , 2002 (mut 6); Kato Hum Mut, 2004; Marsh, Brain, 2009
normal intelligence, had a series of three convulsive seizures between 12 and 17 years of age, but has had none since then	exon1-2 del	no protein produced	50% normal function	mother of the previous case. X-inactivation analysis showed a ratio of 61:39 in blood	Marsh, Brain, 2009
delayed psychomotor development, severe ID, tonic–clonic seizures since 14 mo of age, spastic tetraplegia with left greater than right, generalized dystonia (onset 12 y of age, muscle contractions of the left limb with flexing at the waist), and strabismus. Hyperexcitability, as well as aggressive and autoaggresive behavior. Intrafamilial variability of phenotypes and progression of symptoms together with the age of the patients, especially dystonia and worsening ID.	c.4A>T	p.S2C	60-70% normal function (possible abnormal folding and agregation)	male Patient V:13	Charzewska, Am J Medical Genet, 2013
delayed psychomotor development, severe ID, speaks only a few words, clumsy tiptoe, walking at 18 months of age, lack of expressive speech, focal dystonia (muscle contractions of the right limb) and spasticity of the upper and lower limbs, worse on the right side, hyperexcitability. Abnormal EEG (generalized epileptic alterations), but no seizures. MRI were normal. Intrafamilial variability of phenotypes and progression of symptoms together with the age of the patients, especially dystonia and worsening ID.	c.4A>T	p.S2C	60-70% normal function (possible abnormal folding and agregation)	male Patient V:12 in the same family	Charzewska, Am J Medical Genet, 2013
He had tremor, spasticity, and focal dystonia (contractures in hip and knee joints) since the second day of life. His psychomotor development was delayed; clumsy and tiptoe walking at 18 months of age, first words at 4 years of age. He had dysarthria and was not able to build sentences. Moderate ID. He manifested poor manipulative skills with disturbance of visual motor coordination, hyperexcitability, and aggressive behavior. EEG showed generalized epileptic alterations, although without seizure development. Intrafamilial variability of phenotypes and progression of symptoms together with the age of the patients, especially dystonia and worsening ID.	c.4A>T	p.S2C	60-70% normal function (possible abnormal folding and agregation)	Male Patient V:31 in the same family	Charzewska, Am J Medical Genet, 2013
ID, hyperexcitability and aggressive behavior. He had focal dystonia (contractures in joints) and spasticity of upper limbs, left greater than right. He was unable to speak; his movements were disturbed (clumsy tiptoe walking). Normal EEG and cranial MRI. Intrafamilial variability of phenotypes and progression of symptoms together with the age of the patients, especially dystonia and worsening ID.	c.4A>T	p.S2C	60-70% normal function (possible abnormal folding and agregation)	Male patient IV:6 in the same family	Charzewska, Am J Medical Genet, 2013
ID, hyperexcitability, aggressive behavior, spasticity and focal dystonia (muscle contractions of the left hand). Manifested clumsy, tiptoe walking, was able to speak several words, and had dysarthria and strabismus. His EEG showed generalized epileptic alterations. Intrafamilial variability of phenotypes and progression of symptoms together with the age of the patients, especially dystonia and worsening ID.	c.4A>T	p.S2C	60-70% normal function (possible abnormal folding and agregation)	Male patient IV:16 in the same family	Charzewska, Am J Medical Genet, 2013
asymptomatic	c.4A>T	p.S2C	80-85% normal function	several carrier mothers in the same family	Charzewska, Am J Medical Genet, 2013

ISSX	c.34GT	p.E12* (reinitiation of mRNA translation at p.M41)	~ 40-50% normal function (lacks the OP and decreased level of the protein)	2 brothers	Moey, Eur J Hum Genet, 2016
ISSX/WS	c.81C>G	p.Y27* (reinitiation of mRNA translation at p.M41)	~ 40-50% normal function (lacks the OP and decreased level of the protein)	Patient IV-1 (cousin of Patient IV-2)	Fullston, Eur J Hum Genet, 2010
Ohtahara	c.81C>G	p.Y27* (reinitiation of mRNA translation at p.M41)	~ 40-50% normal function (lacks the OP and decreased level of the protein)	Patient IV-2 (cousin of Patient IV-1)	Fullston, Eur J Hum Genet, 2010
asymptomatic, normal intelligence	c.81C>G	p.Y27* (reinitiation of mRNA translation at p.M41)	70-75% normal function (lacks the OP and decreased level of the protein)	2 carrier mothers	Fullston, Eur J Hum Genet, 2010
Ohtahara + microcephaly (at 2 years old, this boy was suffering from dysmyelination, spastic quadriplegia, and seizures)	c.84C>A	p.C28* (possible reinitiation of mRNA translation at p.M41)	~ 40-50% normal function (lacks the OP and decreased level of the protein)	a 2 year-old boy (and a brother who died)	Zaker, Mol Genet Genom Med, 2024
asymptomatic	c.84C>A	p.C28* (possible reinitiation of mRNA translation at p.M41)	70-75% normal function (lacks the OP and decreased level of the protein)	mother of the two previous boys	Zaker, Mol Genet Genom Med, 2024
X-linked moderate to profound ID, 2 had epilepsy, one had agressive behaviour, all have normal MRI	c.98T>C	p.L33P	~ 80% normal function, decreased interaction with cofactor TLE	9 male patients across two generations (family P8 (= MRX54))	Ben Jemaa, Am J Med Genet, 1999; Bienvenu, Hum Mol Genet, 2002
asymptomatic	c.98T>C	p.L33P	~ 90% normal function, decreased interaction with cofactor TLE	carrier women of the same family	Ben Jemaa, Am J Med Genet, 1999; Bienvenu, Hum Mol Genet, 2002, family P8 (= MRX54)
Severe langage delay, obesity and autistic behaviour	c.112C>T	p.P38S	75-85% normal function	P109 family	Poirier, Neurogenetics, 2006
asymptomatic	c.112C>T	p.P38S	85-92% normal function	carrier mother of the previous case	Poirier, Neurogenetics, 2006
borderline ID with langage delay	c.112C>T	p.P38S	75-85% normal function	grand-father of the previous case	Poirier, Neurogenetics, 2006
XLAG	c.196+2T>C (IVS1+2T>C)	skipping exon 1 (196 pb) or retention of intron 1: loss of translation start or frameshift and early termination, likely resulting in NMD	0% normal function	LR01-330	Kato, Hum Mutation, 2004; Kato and Dobyns, J Child Neurol, 2005
asymptomatic	c.196+2T>C (IVS1+2T>C)	most likely NMD	50% normal function	mother of LR01-330	Marsh, Brain, 2009
XLAG	Exon2_5del	p.G66_C562del (most likely NMD)	0% normal function	LR02-139	Bonneau, Ann Neurol, 2002 (case 2-II.1); Kato, Hum Mut, 2004
developmental delay, short attention span, learning difficulties, problem with balance, complete ACC and a slightly enlarged cisterna magna with a small cerebellar vermis	Exon2_5del	p.G66_C562del (most likely NMD)	50% normal function	sister of LR02-139	Bonneau, Ann Neurol, 2002 (case 2-II.3)

asymptomatic	Exon2_5del	p.G66_C562del (most likely NMD)	50% normal function	mother of LR02-139. X-inactivation analysis showed a ratio of 11:89 in blood, consistent with possibly favorable skewed X-inactivation	Marsh, Brain, 2009
pharmacosensitive focal seizures, mild ID, partial ACC	c.201_204del	p.P68Rfs*99 (most likely NMD)	50% normal function	female patient (*de novo*). X-inactivation analysis showed a ratio of 64:36 in blood	Gras, J Med Genet, 2023 (case 3)
XLAG	c.232G>T	p.E78* (most likely NMD)	0% normal function	LR02-138 (possibly related to LR02-195)	Bonneau, Ann Neurol, 2002 (case 1-II.1); Kato, Hum Mut, 2004; Kato and Dobyns, J Child Neurol, 2005
complete ACC but clinically normal	c.232G>T	p.E78* (most likely NMD)	50% normal function	mother of LR02-138. X-inactivation analysis showed a ratio of 95:05 in blood, consistent with possible favorable skewed X-inactivation	Bonneau, Ann Neurol, 2002 (case 1-I.2)
complete ACC but clinically normal	c.232G>T	p.E78* (most likely NMD)	50% normal function	sister of LR02-138. X-inactivation analysis showed a ratio of 26:74 in blood, consistent with possible favorable partially skewed X-inactivation	Bonneau, Ann Neurol, 2002 (case 1-II.2)
XLAG	c.232G>T	p.E78* (most likely NMD)	0% normal function	LR02-195 (possibly related to LR02-138)	Bonneau, Ann Neurol, 2002 (case 3-III.1); Kato, Hum Mut, 2004
normal intelligence, partial ACC	c.232G>T	p.E78* (most likely NMD)	50% normal function	mother of LR02-195. X-inactivation analysis showed a ratio of 59:41 in blood	Bonneau, Ann Neurol, 2002 (case 3-II.2)
complete ACC, motor development globally delayed, moderate ID, tonic-clonic epilepsy which stopped at 11 years old	c.232G>T	p.E78* (most likely NMD)	50% normal function	aunt of LR02-195. X-inactivation analysis showed a ratio of 83:17 in blood, consistent with possible unfavorable skewed X-inactivation	Bonneau, Ann Neurol, 2002 (case 3-II.3)
XLAG	c.335-368del	delA112_G123fs (likely NMD)	0% normal function	LR01-331	Kato, Hum Mut, 2004; Kato and Dobyns, J Child Neurol, 2005
asymptomatic	c.335-368del	delA112_G123fs (likely NMD)	50% normal function	carrier mother of the previous case. X-inactivation analysis showed a ratio of 62:38 in blood	Marsh, Brain, 2009
XLAG	c.392-452del	delP131_A151fs (most likely NMD)	0% normal function	LR02-320 (*de novo*)	Kato, Hum Mut, 2004; Kato and Dobyns, 2005
XLAG	c.420-451del(32pb)	p.G141fs*85 (most likely NMD)	0% normal function	Patient 1	Ogata, Am J Med Genet, 2000; Kitamura, Nat Genet, 2002 (mut 1); Kato, Hum Mut, 2004; Kato and Dobyns, 2005
moderate ID, DEE, partial ACC, pharmacosensitive epilepsy, neurological findings	c.487C>T	p.Q163* (most likely NMD)	50% normal function	a female patient (de novo) (Case 10)	Gras, J Med Genet, 2023
XLID, moderate to profund ID, cerebellar symptoms in 2/6 patients, 5/6 patients had epilepsy, 3/6 patients were irritable and agressive	c.488A>G	p.Q163R	60-80% normal function?	Family T4	Bienvenu, Hum Mol Genet, 2002
moderate ID, ASD, complete ACC, pharmacosensitive epilepsy, orolingual dyspraxia and fine motor skills impaired	c.518dupA	p.S174Vfs*64 (most likely NMD)	50% normal function	a female patient (*de novo*) (Case 9)	Gras, J Med Genet, 2023
moderate ID, ASD + ADHD, complete ACC, no epilepsy, poor coordination	c.521C>A	p.S174* (most likely NMD)	50% normal function	a female patient (Case 5) (*de novo*). X-inactivation analysis showed a ratio of 73:27 in blood, consistent with a partly skewed X-inactivation.	Gras, J Med Genet, 2023

XLAG and HYD	c.617delG	p.G206Afs*119 (most likely NMD)	0% normal function	LR01-038a1 (III-3) and LR01-038a3 (III-2) (2 brothers)	Kato, Hum Mut, 2004; Kato and Dobyns, J Child Neurol, 2005; Marsh, Brain, 2009
asymptomatic	c.617delG	p.G206Afs*119 (most likely NMD)	50% normal function	carrier mother and grandmother of previous cases. X-inactivation analysis in the mother showed a ratio of 67:33 in blood	Kato, Hum Mut, 2004; Marsh, Brain, 2009
moderate ID, DDE, partial ACC, hypotonia	c.617delG	p.G206Afs*119 (most likely NMD)	50% normal function	carrier sister of family LR01-038 (III-5). X-inactivation analysis showed a ratio of 29:71 in blood	Kato, Hum Mut, 2004; Marsh, Brain, 2009
XLAG	c.619-647del	delV207_A216fs (most likely NMD)	0% normal function	LR02-311a2	Kato, Hum Mut, 2004; Kato and Dobyns, J Child Neurol, 2005
Schizophrenia	not indicated	p.R264Q	uncharacterized effect on the resulting protein	Female	Nakamura, Brain Behav Immun, 2019
XLAG	c.790delC	p.R264Gfs*60 (most likely NMD)	0% normal function	LP97-107 (*de novo*)	Kitamura, Nat Genet, 2002 (mut 2); Kato, Hum Mut, 2004; Kato and Dobyns, J Child Neurol, 2005
XLAG	c.790delC	p.R264Gfs*60 (most likely NMD)	0% normal function	one male case (Family 1)	Uyanik, Neurol, 2003
asymptomatic but partial posterior ACC with enlarged ventricles	c.790delC	p.R264Gfs*60 (most likely NMD)	50% normal function	the carrier mother (Family 1)	Uyanik, Neurol, 2003
ASD	no information	p.A279T	uncharacterized effect on the resulting protein	Female patient	Nakamura, Brain Behav Immun, 2019
X-linked moderate ID, 2/4 male patients had epilepsy, macrocephaly, scolisosis, hyertelorism, no brain malformation	c.856G>A	p.G286S	60-80% normal function?	patient P25	Bienvenu, Hum Mol Genet, 2002
XLAG	c.862del	p.E288Sfs*37 (most likely NMD)	0% normal function	boy	Li, J Med Genet, 2024
asymptomatic but ACC	c.862del	p.E288Sfs*37 (most likely NMD)	50% normal function	the mother of the previous case	Li, J Med Genet, 2024
severe ID, DEE (started at 1 mo old), pharmacoresistant multiple seizure types, spastic dystonic quadriplegia and severe developmental delay, microcephaly	c.869C>A	p.S290* (most likely NMD)	50% normal function, possible unfavorable skewed X-inactivation of the wild-type allele in brain?	chinese Female patient (*de novo*)	Kwong, PLoS One, 2015
partial ACC, mild difficulties in fine motor skills and global attention, especially in the expressive language domain	c.922G>T	p.E308* (most likely NMD)	50% normal function	Female patient. X-inactivation analysis showed a ratio of 43:57 in blood	Traversa, Mol Genet Genomic Med, 2020
XLAG	c.980delAACA	p.K327fs (most likely NMD)	0% normal function	male patient (*de novo*)	Miyata, Brain Dev, 2009
XLAG	c.982delCinsTTT	p.Q328Ffs*37 (most likely NMD)	0% normal function	male patient	Mattiske, Hum Mut, 2017
Mild ID, Seizures, ACC	c.982delCinsTTT	p.Q328Ffs*37 (most likely NMD)	50% normal function	carrier sister of the previous case. No skewed X-inactivation in blood	Mattiske, Hum Mut, 2017
mild ID, partial seizures, ACC	c.982delCinsTTT	p.Q328Ffs*37 (most likely NMD)	50% normal function	carrier mother of the previous cases	Mattiske, Hum Mut, 2017
WEST, dystonia + mitochondrial dysfunction	c.989G>A	p.R330H	50-60% normal function?	3 males (a 13-year old Chinese boy and his 2 brothers)	Kwong, Brain Dev, 2019
asymptomatic	c.989G>A	p.R330H	75-80% normal function	sister and mother of the previous cases	Kwong, Brain Dev, 2019
suspected mitochondrial disease	c.989G>A	p.R330H	50-60% normal function?	Patient 14	Tsang, Human Genomics, 2020

WEST	c.989G>A	p.R330H	50-60% normal function?		Krey, Eur J Pediatric Neurol, 2020
XLAG	c.994C>T	p.R332C	0% normal function	one male case in Family 2	Uyanik, Neurol, 2003
XLAG with a milder phenotype	c.995G>C	p.R332P	10% normal function	LR00-052	Kato, Hum Mut, 2004; Kato and Dobyns, J Child Neurol, 2005
asymptomatic	c.995G>C	p.R332P	55% normal function	mother of LR00-052	Marsh, Brain, 2009
XLAG with a milder phenotype than premature termination mutations	c.995G>A	p.R332H	10% normal function	LR00-023	Kitamura, Nat Genet, 2004 (case 3); Kato and Dobyns, J Child Neurol, 2005
asymptomatic	c.995G>A	p.R332H	55% normal function	mother of LR00-023. X-inactivation analysis showed a ratio of 33:67 in blood	Marsh, Brain, 2009
XLAG		p.R332L	0% normal function	Clinvar ID:157765	
XLAG with a milder phenotype than premature termination mutations, ACC	c.996/1001del9	delT333/T334_T335/336	10% normal function	CMS3242	Bhat, Am J Med Genet, 2005
Proud/ACC (moderate to severe mental retardation with ambiguous or hypoplastic male genitalia)	c.998C>A	p.T333N	20% normal function	LR02-083a1/a2/a3 (3 relative males)	Proud, Am J Med Genet, 1992 (Cases III.2, III.5 and IV.1 Fig 1C); Kato, Hum Mut, 2004
asymptomatic, partial ACC	c.998C>A	p.T333N	55% normal function?	mother of the three previous cases	Marsh, Brain, 2009; Proud, Am J Med Genet, 1992
severe ID, DEE, ACC, spastic quadriplegia	c.998C>A	p.T333N	55% normal function? Possible unfavorable skewed X-inactivation of the wild-type allele in brain?	aunt of the previous case	Proud, Am J Med Genet, 1992 ; Kato, Hum Mut, 2004
asymptomatic	c.998C>A	p.T333N	55% normal function	other aunt of the previous case	Proud, Am J Med Genet, 1992 ; Kato, Hum Mut, 2004
mild ID	c.998C>A	p.T333N	55% normal function	cousin of the previous case	Proud, Am J Med Genet, 1992 ; Kato, Hum Mut, 2004
cerebral atrophy at MRI, hypsarrythmia at EEG		p.T333S	70-80% normal function (decreased amount prot)?	LR03-413	Mirzaa, Pediatr Neurol, 2013
XLAG (small penis and retention of testes at birth, and he had intractable seizures and chronic diarrhea from birth. He evidenced no developmental milestone in his life. Brain MRI showed thick and smooth brain surface with agenesis of corpus callosum, hypoplasia of thalami, and large occipital cyst. At 10 months of age, he had severe growth retardation, such as, a weight of 3,456 g (¡6.4 SD) and height of 61.0 cm (¡4.9 SD). He died of central respiratory failure at 11 months of age)	c.1013delAinsCC	p.Y338Sfs*194 (NMD?)	0% normal function	a japanese male patient	Okazaki, Acta Neuropathol, 2008
IQ = 70, her brain MRI demonstrated focal cortical dysplasia in the left temporal medial region, normal formation of CC	c.1013delAinsCC	p.Y338Sfs*194 (NMD?)	50% normal function?	mother of the previous case	Okazaki, Acta Neuropathol, 2008
ID, DEE (started at 11 mo), pharmacosensitive epilepsy, ACC, cortical dysplasia of the right temporal medial region	c.1013delAinsCC	p.Y338Sfs*194 (NMD?)	50% normal function?	sister of the previous case	Okazaki, Acta Neuropathol, 2008
ACC but normal development, no seizures	c.1013-1019dupACCAGCT	p.E341Pfs*193 (NMD?)	50% normal function ?	female fœtus	Devi, Cureus, 2023

Asymptomatic, partial ACC	c.1013-1019dupACCAGCT	p.E341Pfs*193 (NMD?)	50% normal function?	mother of the previous girl	Devi, Cureus, 2023
XLAG with a milder phenotype than premature termination mutations	c.1028T>A	p.L343Q	10% normal function	LR02-262a1, LR02-262a2 = 2 brothers	Kitamura, Nat Genet, 2002 (mut8/9); Kato, Hum Mut, 2004; Kato and Dobyns, J Child Neurol, 2005
asymptomatic	c.1028T>A	p.L343Q	55% normal function	mother of the 2 previous brothers	Marsh, Brain, 2009
XLAG	c.1034G>C	p.R345P	0% normal function	MDC1009 (*de novo*)	Gonzalez-Moron, PLoS One, 2017
XLAG, with a milder phenotype than premature termination mutations	c.1058C>G	p.P353R	0% normal function	LR03-015 (family)	Kato, Hum Mut, 2004
XMESID (X-linked myoclonic epilepsy, generalized spasticity and severe ID)	c.1058C>T	p.P353L	60% normal function?	Australian family, 6 males	Stromme, Nat Genet, 2002; Scheffer, Neurol, 2002
subtle hyperrflexia (+ late-onset spastic ataxia in one woman)	c.1058C>T	p.P353L	80% normal function?	female carriers of the Australian family	Stromme, Nat Genet, 2002; Scheffer, Neurology, 2002
ACC, severe ID, infantile spasms and subsequent tonic seizures, spastic/dyskinetic quadriparesis, severe limb contractures, scoliosis, general dystonia, absent speech. Ressembles Proud syndrome	c.1072A>T	p.R358W	40-50% normal function (decreased DNA binding)?	a 23-year-old man who died at 25	Conti, Am J Med Genet, 2011
asymptomatic	c.1072A>T	p.R358W	80% normal function ?	Mother of the following case	Conti, Am J Med Genet, 2011
ISSX, ACC and widespread neuronal migration disorder	c.1074G>T	p.R358S	50% normal function (decreased DNA binding)?	Family D	Fullston, Clinical Genetics, 2011
learning difficulties	c.1074G>T	p.R358S	75% normal function (decreased DNA binding)?	carrier female of Family D	Fullston, Clinical Genetics, 2011
HYD/AG	c.1105G>T	p.E369* (most likely NMD)	20-30% normal function?	LR02-162 (*de novo*)	Kato, Hum Mut, 2004
microcephaly, ISSX emerging into tonic-clonic and a Lennox-Gastaut pattern, ACC with subtle loss of white matter volume in left cerebral hemisphere, hypotonia, stridor, and dysmorphic features	c.1105G>A	p.E369K	40-50% normal function?	a 12 mo male patient (*de novo*)	this study
severe ID and ASD without seizures or brain malformations, mildly delayed motor and severely delayed speech development	c.1109C>T	p.A370V	80% normal function ?	one male patient	Thai, Hum Mutation, 2020
asymptomatic	c.1109C>T	p.A370V	90% normal function ?	carrier mother of the previous case	Thai, Hum Mutation, 2020
severe (pharmacoresistant generalized seizures + febrile seizures, DEE, complete ACC, severe ID, dystonia left side)	c.1111C>T	p.R371* (most likely NMD)	50% normal function but possible GOF effects if NMD not 100% efficient	a female patient (case 2) (*de novo*). X-inactivation analysis showed a ratio of 45:55 in blood.	Gras, J Med Genet, 2023
Seizure onset was at 4 do with predominant seizure types including myoclonic seizures, which occurred in clusters as well as sporadically, and focal tonic seizures, CC thinning, profound axial hypotonia and weakness, severe developmental delay. At 10 months of age, he developed intermittent focal and segmental dystonia, involving mostly the distal extremities, including prominent hand dystonia.	c.1111C >G	p.R371G	60-70% normal function?	a male patient	Akula, Annals Clin Transl Neurol, 2024

ASD for all, moderate to severe ID, none to severe seizures	c.1112G>A	p.R371Q	80% normal function?	4 male patients in the same family	Thai, Hum Mutation, 2020
asymptomatic	c.1112G>A	p.R371Q	90% normal function ?	carrrier females of the previous family	Thai, Hum Mutation, 2020
XLAG	c.1117C>T	p.Q373* (most likely NMD)	0% normal function	LR00-175	Kitamura, Nat Genet, 2002 (mut4); Kato, Hum Mut 2004; Kato and Dobyns, J Child Neurol, 2005
asymptomatic	c.1117C>T	p.Q373* (most likely NMD)	50% normal function	mother of LR00-175	Marsh, Brain, 2009
XLAG	c.1119+1G>C (IVS3+1G>C)	skipping exon 3 (46 bp) or retention intron 3 (most likely NMD)	0% normal function	LR02-479a1 and LR02-479a2 (2 male relatives)	Kato, Hum Mut, 2004; Kato and Dobyns, J Child Neurol, 2005
Severe (DEE, pharmacoresistant seizures, partial ACC, severe ID, hypotonia)	c.1120G>T	p. V374F	50% normal function but possible DN effects?	a female patient (case 6) (*de novo*)	Gras, J Med Genet, 2023
severe (severe ID, stereotypie, DEE, complete ACC, gyration abnormalities, pharmacosensitive seizures, unable to walk)	c.1124G>T	p.W375L	50% normal function but possible DN effects?	a female patient (Case 8) (*de novo*)	Gras, J Med Genet, 2023
Primary immunodeficiency, severe intractable diarrhea, pneumonia and dysmorphic features	c.1124G>A	p.W375* (most likely NMD)	50% normal function but possible DN effects if NMD not 100% efficient?	a female patient of one year old (*de novo*)	Dr Luis Alberto Pedroza, personnal communication,
severe ID, ISSX, intractable epilepsy,spastic tetraparesis	c.1135C>A	p.R379S (aberrant transport to the nucleus)	40-50% normal function (decreased amount prot in nucleus)	LR07-074	Marsh, Brain, 2009; Shoubridge, Hum Mut, 2010
asymptomatic	c.1135C>A	p.R379S (aberrant transport to the nucleus)	70-75% normal function	Mother of LR07-074. X-inactivation analysis showed a ratio of 5:95 in blood, compatible with a favorable skewed X inactivation	Marsh, Brain 2009
learning disabilities (but no ID), pervasive developmental disorder (ASD) and absence epilepsy	c.1135C>A	p.R379S (aberrant transport to the nucleus)	70-75% normal function	female cousin (LR07-074a1). X-inactivation analysis showed a ratio of 54:46 in blood.	Marsh, Brain 2009
asymptomatic	c.1135C>A	p.R379S (aberrant transport to the nucleus)	70-75% normal function	aunt of LR07-074. X-inactivation analysis showed a ratio of 21:79 in blood, compatible with a favorable skewed X inactivation	Marsh, Brain 2009
XLAG	c.1136G>T	p.R379L (aberrant transport to the nucleus)	0% normal function		Shoubridge, Pathogenetics, 2010
XLAG with other multisysteme manifestations (intestinal manifestations) that were as life limiting as the CNS features. Severe chronic diarrhea resulted in failure to thrive, dehydration, electrolyte derangements, long-term hospitalization, and prompted transition to palliative care. Other multisystem manifestations included megacolon, colitis, pancreatic insufficiency hypothalamic dysfunction, hypothyroidism, and hypophosphatasia	c.1136G>T	p.R379L (aberrant transport to the nucleus)	0% normal function	a baby male (*de novo*)	Coman, Chil Neurol Open, 2017
severe (DEE, pharmacoresistant seizures, complete ACC, severe ID, gyration abnormalities)	c.1139G>A	p.R380Q	50% normal function but possible DN effects?	female patient (case 4) (*de novo*)	Gras, J Med Genet, 2023

EIEE/Ohtahara (ambigous genitalia, profound global developmental delay and intractable tonic seizures since day 1 of life, with a suppression-burst EEG pattern, progound hypotonia, abnormal MRI)	c.1150C>T	p.R384C (aberrant transport to the nucleus)	25-40% normal function (decreased amount prot in nucleus)	one male patient (de novo)	Thai, Hum Mutation, 2020
asymptomatic	c.1150C>T	p.R384C (aberrant transport to the nucleus)	60-70% normal function (decreased amount prot in nucleus)	mother of the previous case	Thai, Hum Mutation, 2020
ID, developmental delay, ACC and DDE	c.1153A>T	p.K385* (most likely NMD)	50% normal function but possible skewed X-inactivation in the brain?	a female patient	Han, IJMS, 2024
Mild ID with a need for assistance with complex tasks, no seizures, dysgenesis of CC	c.1153A>T	p.K385* (most likely NMD)	50% normal function	mother of the previous case	Han, IJMS, 2024
QI = 75, no seizure, no defect of the CC	c.1153A>T	p.K385* (most likely NMD)	50% normal function	grand-mother of the previous case	Han, IJMS, 2024
XLAG	c.1187_1188insC	p.G397Wfs134 (likely escapes NMD but possible unstable protein?)	0% normal function	LR00-185	Kitamura, Nat Gen, 2004 (mut5); Kato, Hum Mut, 2004; Kato and Dobyns, J Child Neurol, 2005
asymptomatic	c.1187_1188insC	p.G397Wfs134 (likely escapes NMD but possible unstable protein?)	50% normal function	mother of LR00-185. X-inactivation analysis showed a ratio of 79:21 in blood.	Marsh, Brain, 2009
partial ACC, ASD, gestual dyspraxia	c.1191del	p.L398Cfs*65 (most likely NMD)	50% normal function	a female patient (Case 7) (*de novo*)	Gras, J Med Genet, 2023
ASD, global development delay, ACC, absence and focal seizures (onset 10 mo age), cerebral atrophy, and a left parafalcine cyst	c.1206del	p.P403Rfs*60 (most likely NMD)	50% normal function	indonesian Female patient	Iskandar, Annals Med Surg, 2023
Pharmacoresistant epilepsy, ISSX, DEE, complete ACC, severe ID, pyramidal syndrome	c.1349C>A	p.S450* (most likely NMD)	50% normal function but possible skewed X-inactivation in brain?	a female patient (Case 1) (*de novo*). X-inactivation analysis showed a ratio of 63:37 in blood.	Gras, J Med Genet, 2023
XLAG	c.1372delG	p.A458Rfs*4 (most likely NMD)	0% normal function	LP95-060 (*de novo*)	Dobyns, Am J Med Genet, 1999 (case 2); Kato, Hum Mut, 2004; Kato and Dobyns, J Child Neurol, 2005
XLAG	c.1372delG	p.A458Rfs*4 (most likely NMD)	0% normal function	LR01-102	Kitamura, Nat Genet, 2002 (mut7); Kato and Dobyns, J Child Neurol, 2005
asymptomatic	c.1372delG	p.A458Rfs*4 (most likely NMD)	50% normal function	mother of the previous case	Marsh, Brain, 2009
Developmental and epileptic encephalopathy 1 (HP:0001250 Seizures;HP:0006872 Cerebral hypoplasia)	c.1399G>T	p.G467* (most likely NMD)	50% normal function but possible skewed X-inactivation in the brain?	female baby (P19) (*de novo*)	Chuan, Front Genet, 2022
severe (profound developmental delay, West (started at 9 mo), DEE, pharmacoresistant epilepsy (tonic clonic, gelastic, absence and focal seizures), partial ACC, quadriplegic Cerebral Palsy, significant spasticity of her 4 members, choreoathetoid movements)	c.1406_1415del	p.A469Dfs*20 (likely escapes NMD)	50% normal function but possible skewed X-inactivation in the brain? Other reason?	1 of 2 female monozygotic twins (Twin 2). X-inactivation analysis showed a ratio of 46:40 in blood (75:25 in hair follicle)	Rodgers, Eur J Med Genet, 2021

a bit less severe than her sister (developmental delay, ISS (started at 14 mo), DEE, ASD, cerebral palsy, pharmacoresistant epilepsy (focal discognitive seizures), hypoplastic CC, not able to say words, able to walk)	c.1406_1415del	p.A469Dfs*20 (likely escapes NMD)	50% normal function but possible skewed X-inactivation in the brain? Other reason?	1 of 2 female monozygotic twins (twin 1). X-inactivation analysis showed a ratio of 60:40 in blood (56:44 in hair follicle)	Rodgers, Eur J Med Genet, 2021
XLAG, ACC, intractable epilepsy, and abnormal genitalia, died at 17 mo old, no psychomotor development	c.1419_1420insAC	p.H473fs (likely escapes NMD)	0% normal function	a boy	Hartmann, Neuropediatrics, 2004
ACC, intractable epilepsy, and abnormal genitalia, died at 18 mo old, no psychomotor development	c.1419_1420insAC	p.H473fs (likely escapes NMD)	0% normal function	brother of the previous case	Hartmann, Neuropediatrics, 2004
XLAG	c.1427-1428_delTCinsAA	p.F476* (likely escapes NMD)	0% normal function	MDC1039 (de novo)	Gonzalez-Moron, PLoS One, 2017
Severe ID, speech impairment, testicular dysgenesis	c.1449-1-1456del GGCTCTTTT	p.R483Sfs46* (likely escapes NMD)	50-70% normal function (absence aristaless domain)	3 brothers (family MRID 149)	Rasheed, BMC Med Genomics, 2021
asymptomatic	c.1449-1-1456del GGCTCTTTT	p.R483Sfs46* (likely escapes NMD)	75-80% normal function	mother of the 3 previous cases	Rasheed, BMC Med Genomics, 2021
ISSX/West syndrome, severe ID	IVS4-816_E*5701del	p.R483fs (likely escapes NMD)	50-70% normal function (absence domaine aristaless)	famille norvégienne, 2 males	Stromme, Nat Genet, 2002; Stromme, Brain Dev, 2002
DEE1 (HP:0001250 Seizures;HP:0000574 Thick eyebrow;HP:0001263 Global developmental delay;HP:0001265 Hyporeflexia;HP:0008897 Postnatal growth retardation;HP:0001641 Abnormality of the pulmonary valve;HP:0001601 Laryngomalacia;HP:0002090 Pneumonia)	c.1441_1447dup	p.R483Ifs*51 (likely escapes NMD)	50-70% normal function (absence domaine aristaless)	male baby (P125)	Chuan, Front Genet, 2022
ISSX	c.1448+1G>A	truncation without exon 5 (likely escapes NMD)	50-70% normal function (absence domaine aristaless)	male	Takeshita, Hum Gen Var, 2020
developmental and epileptic encephalopathy	c.1449-1G>C	p.L484* (likely escapes NMD)	50-70% normal function (absence domaine aristaless)	3 males	Shoubridge, Am J Med Genet, 2019
early onset epileptic encephalopathy (DEE) (3 mo), severe global delay, ASD, pharmacoresistant epilepsy, significantly ataxic,very poor gross and fine motor skills, no brain malformation and normal CC	c.1459delA	p.T487Qfs*5 (likely escapes NMD)	75-80% normal function but possible skewed X-inactivation in the brain?	Female patient (*de novo*), X-inactivation analysis showed a ratio of 70:30 in blood consistent with a partially skewed X-inactivation	Bettella, Clin Genet, 2013
developmental delay, ISSX, pharmacoresistant epilepsy, drooling, mild hypotonia, moderate to severe ID	c.1465delG	p.A489Pfs*3 (likely escapes NMD)	70-75% normal function but possible skewed X-inactivation in the brain?	Female patient LR06-362. X-inactivation analysis showed a ratio of 77:23 in blood compatible with a partially skewed X-inactivation.	Wallerstein, Clin Neurol Neurosurgery, 2008 (Case 1)
EIEE/Ohtahara (ambigous genitalia, profound global developmental delay and intractable tonic seizures since day 1 of life, with a suppression-burst EEG pattern, profound hypotonia, abnormal MRI)	c.1471_1472insC	p.L491Pfs*41 (likely escapes NMD)	35-50% normal function (function of aristaless domain affected)	7-month-old boy	Eksioglu, Epilepsia, 2011
anxiety, depression and learning difficulties	c.1471_1472insC	p.L491Pfs*41 (likely escapes NMD)	70-75% normal function (function of aristaless domain affected)	mother of the previous case	Eksioglu, Epilepsia, 2011

schizophrenia with auditor and visual hallucinations and learning disabilities	c.1471_1472insC	p.L491Pfs*41 (likely escapes NMD)	70-75% normal function (function of aristaless domain affected)	maternal aunt of the previous case (II-5)	Eksioglu, Epilepsia, 2011
depression	c.1471_1472insC	p.L491Pfs*41 (likely escapes NMD)	70-75% normal function (function of aristaless domain affected)	2nd maternal aunt of the previous case (II-8)	Eksioglu, Epilepsia, 2011
generalized epilepsy, learning diability and cognitive delay	c.1471_1472insC	p.L491Pfs*41 (likely escapes NMD)	70-75% normal function (function of aristaless domain affected)	maternal half-aunt of the previous case	Eksioglu, Epilepsia, 2011
anxiety, depression and mild cognitive delay	c.1471_1472insC	p.L491Pfs*41 (likely escapes NMD)	70-75% normal function (function of aristaless domain affected)	Maternal grandmother	Eksioglu, Epilepsia, 2011
sudden infant death	c.1489G>T	p.A497S	effect in heart?	boy of 2 months and a half	this report
XLAG with a milder phenotype than premature termination mutations, cerebellar hypoplasia, congenital microcephaly, complete agyria, and ACC, basal ganglia appeared normal	c.1561G>A	p.A521T		LR02-264 (sporadic)	Kato, Hum Mut, 2004; Kato and Dobyns, J Child Neurol, 2005
Ohtahara that evolved into WS, severe scoliosis, mild undescended testes with a normal penis, severe spastic quadriplegia, profound ID, and generalized tonic seizures	c.1564-1568dupACGGC	p.A524Rfs*11 (likely escapes NMD)	35-50% normal function (function of aristaless domain affected)	a japanese boy	Kato, Epilepsia, 2010
asymptomatic	c.1564-1568dupACGGC	p.A524Rfs*11 (likely escapes NMD)	70-75% normal function (function of aristaless domain affected)	mother of the previous japanese boy	Kato, Epilepsia, 2010
ISSX	c.1579_1582delAGAC	p.R527fs*5 (likely escapes NMD)	50% normal function (function of aristaless domain affected)	?	Tumiene, Clin Genet, 2018
EIEE/West	c.1600G>C	p.A534P	35-50% normal function (function of aristaless domain affected)	a chinese boy	Arafat, Sci Rep, 2017
Ohtahara (EIEE) then developed status dystonicus (=generalized dystonia)	c.1600G>C	p.A534P	35-50% normal function (function of aristaless domain affected)	a boy	Gorman, Eur J Paediatric Neurol, 2018
Ohtahara syndrome OS that evolved into West syndrome phenotype and subsequently into an epileptic encephalopathy with polymorphic and refractory seizures	c.1604T>A	p.L535Q	35-50% normal function (function of aristaless domain affected)	2 boys of the same family	Giordano, Am J Med Genet, 2010
asymptomatic	c.1604T>A	p.L535Q	70-75% normal function (function of aristaless domain affected)	3 carrier mothers	Giordano, Am J Med Genet, 2010

XLID	C>A	p.R536S	75-80% normal function (function of aristaless domain affected)	a male patient	Hu, Mol Psychiatry 2016
tonic spasms that occurred in clusters associated with abnormal EEG 2 months after birth, cerebral palsy	c.1604-1605insT	p.R536Efs*137	35-50% normal function (function of aristaless domain affected)	a boy (uncle to the 2 following patients)	Kato, Epilepsia, 2010
Ohtahara that evolved into WS. At the age of 13 years, he showed marked growth failure, microcephaly, severe spastic quadriplegia with no voluntary movements; myoclonic seizures or tonic seizures, which occurred as a single seizure or as clusters of seizures 10–20 times a day; no social contact	c.1604-1605insT	p.R536Efs*137	35-50% normal function (function of aristaless domain affected)	2 brothers	Kato, Epilepsia, 2010
asymptomatic	c.1604-1605insT	p.R536Efs*137	70-75% normal function (function of aristaless domain affected)	mother of the two previous boys	Kato, Epilepsia, 2010
Ohtahara-West		p.L537Q	35-50% normal function (function of aristaless domain affected)		Sartori, J Child Neurol, 2011
XLID with abnormal genitalia	c.1614G>T	p.K538N	60-70% normal function (function of aristaless domain affected)?	a boy	Sirisena, Sex Dev, 2014
Ohtahara	c.1616C>A	p.A539D	35-50% normal function (function of aristaless domain affected)	1 boy (III-3)	Tapie, Clin Cas Rep, 2017
mild ID	c.1616C>A	p.A539D	70-75% normal function (function of aristaless domain affected)	carrier mother of III-3 and following case	Tapie, Clin Cas Rep, 2017
ISSX started at 19 days old, intractable epileptic encephalopathy syndrome, he died at 5 yo.	c.1616C>A	p.A539D	35-50% normal function (function of aristaless domain affected)	half-brother of III-3 (from the same mother)	Tapie, Clin Cas Rep, 2017
