# Supplementary material for: Cellular Functional Analyses of ARX Variants Reveal New Insights Into Genotype–Phenotype Correlations in Neurodevelopmental Disorders Among Male and Female Patients
Source: Hum Mutat. 2026 Apr 7;2026:4732622. doi: 10.1155/humu/4732622 (PMC13058441; doi:10.1155/humu/4732622)
Supplement: Supplementary file 3 — Supporting Information 3 Figure S1 presents Western‐blot analysis showing a faint band (indicated by an arrow) corresponding to a longer peptide likely produced by RNA editing of the variant or stop codon readthrough of the variant p.S174V∗fs64. The blot was detected using a polyclonal antibody directed against ARX homeodomain. Figure S2 presents Transcriptional activation capacity of the different ARX variants in 293T cells. Plasmids encoding WT‐ARX or mutant constructs were transfected in 293T cells, and their capacity to activate the expression of the luciferase reporter gene under the control of Lmo1 promoter was assessed. (a) Several truncation mutations as well as missense variants located outside ARX homeodomain caused severe loss‐of‐function (represented in light gray). (b) In contrast, missense or truncation variants located within or in close proximity but downstream ARX homeodomain caused an apparent gain‐of‐function (represented in dark gray) with an overactivation of the expression of the reporter gene under the control of Lmo1 promoter region. (a–b) Comparison of each condition with WT‐ARX, one‐way ANOVA with Dunnett′s post hoc test. ∗, p < 0.05; ∗∗, p < 0.01; ∗∗∗, p < 0.001; and ∗∗∗∗, p < 0.0001. Truncation variants are represented in dark gray whereas missense variants are in light gray. (c) Schematic representation of the variants that showed an apparent gain‐of‐function in luciferase experiments. Figure S3 presents several variants exhibit similar immunolabeling patterns to WT‐ARX in N2a Cells. N2a cells were transfected with plasmids encoding myc‐expressing variants, that were detected by immunofluorescence with anti‐myc antibody. N2a cell nuclei were stained with DAPI. Several variants had similar pattern of subcellular expression as WT‐ARX with a majority of diffuse nuclear staining and occasional faint cytoplasmic labeling. Scale bar: 10 μm. Figure S4 presents Transcriptional repression capacity of the different ARX variants in the presence of [file HUMU-2026-4732622-s003.pptx]

## Slide 1
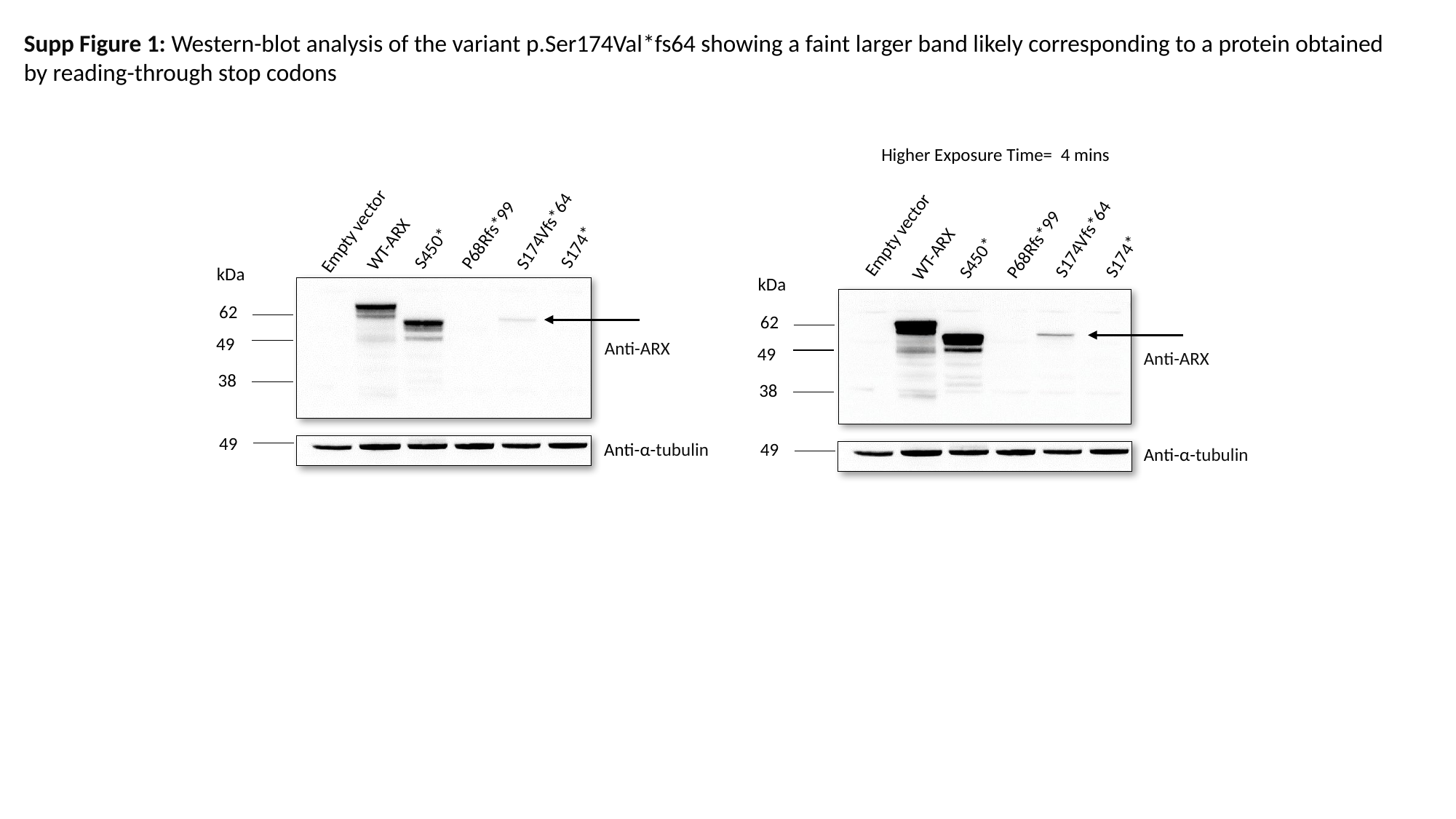

Supp Figure 1: Western-blot analysis of the variant p.Ser174Val*fs64 showing a faint larger band likely corresponding to a protein obtained by reading-through stop codons
Higher Exposure Time= 4 mins
S174Vfs*64
S174Vfs*64
Empty vector
Empty vector
P68Rfs*99
WT-ARX
P68Rfs*99
S174*
WT-ARX
S450*
S174*
S450*
kDa
kDa
62
62
49
Anti-ARX
49
Anti-ARX
38
38
49
49
Anti-α-tubulin
Anti-α-tubulin

## Slide 2
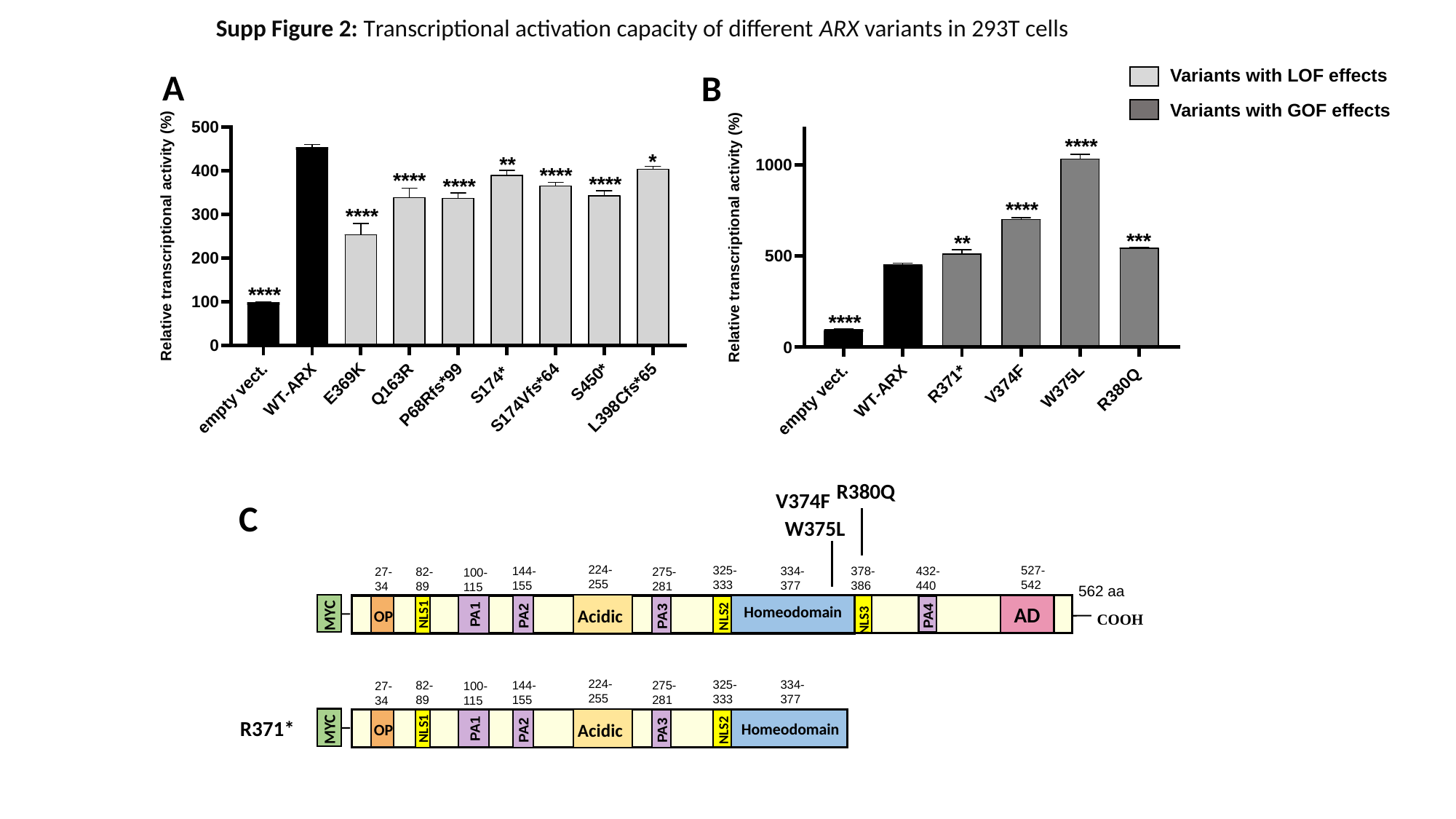

Supp Figure 2: Transcriptional activation capacity of different ARX variants in 293T cells
Variants with LOF effects
A
B
Variants with GOF effects
R380Q
 V374F
 W375L
C
224-255
527-542
325-333
334-377
378-386
432-440
144-155
275-281
82-89
27-34
100-115
NLS1
562 aa
PA4
Acidic
AD
PA3
PA2
OP
PA1
NLS2
COOH
NLS3
MYC
Homeodomain
224-255
325-333
334-377
144-155
275-281
82-89
27-34
100-115
NLS1
Acidic
PA3
PA2
OP
PA1
NLS2
MYC
Homeodomain
R371*

## Slide 3
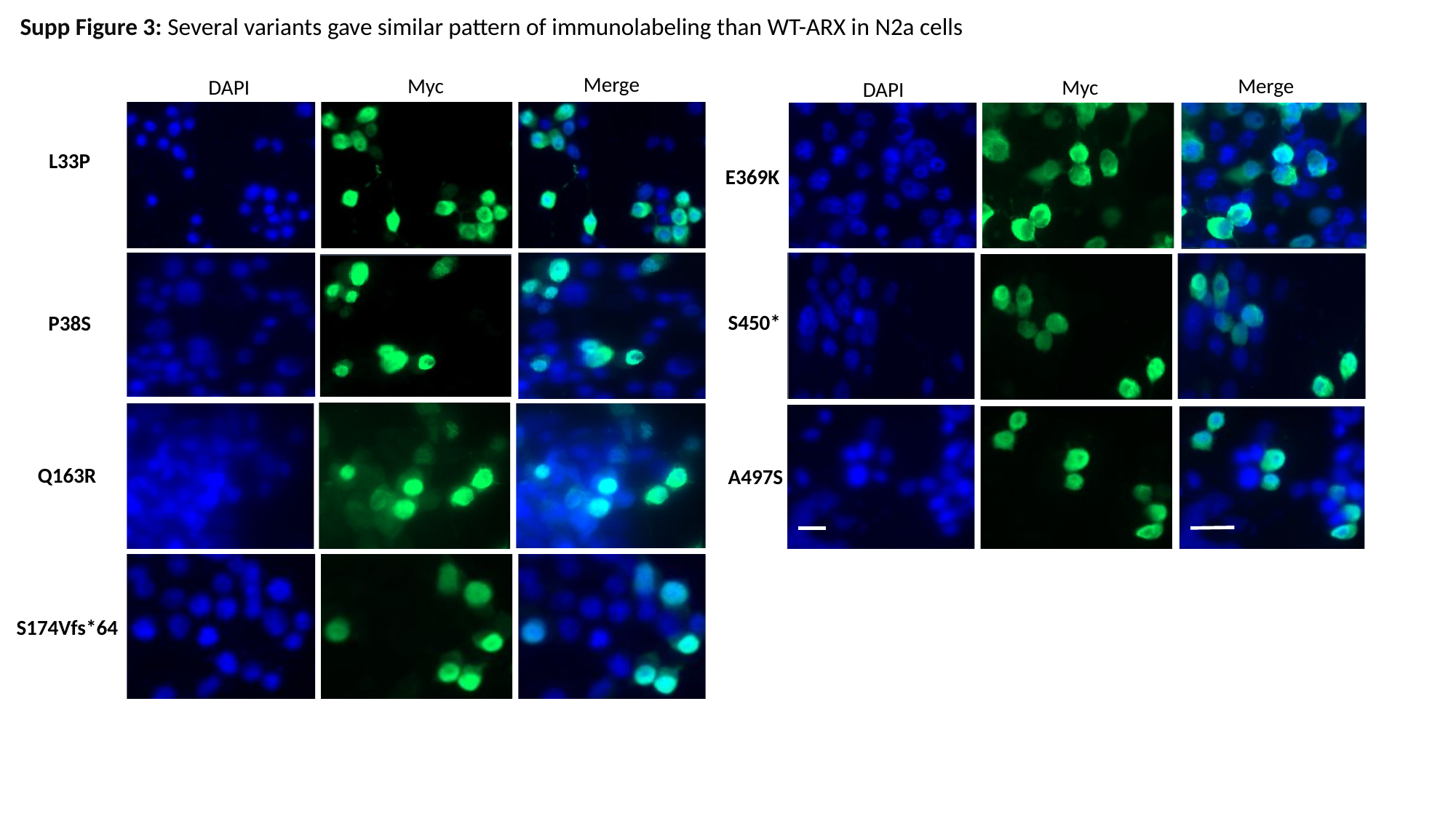

Supp Figure 3: Several variants gave similar pattern of immunolabeling than WT-ARX in N2a cells
Merge
Myc
Merge
Myc
DAPI
DAPI
L33P
E369K
S450*
P38S
Q163R
A497S
S174Vfs*64

## Slide 4
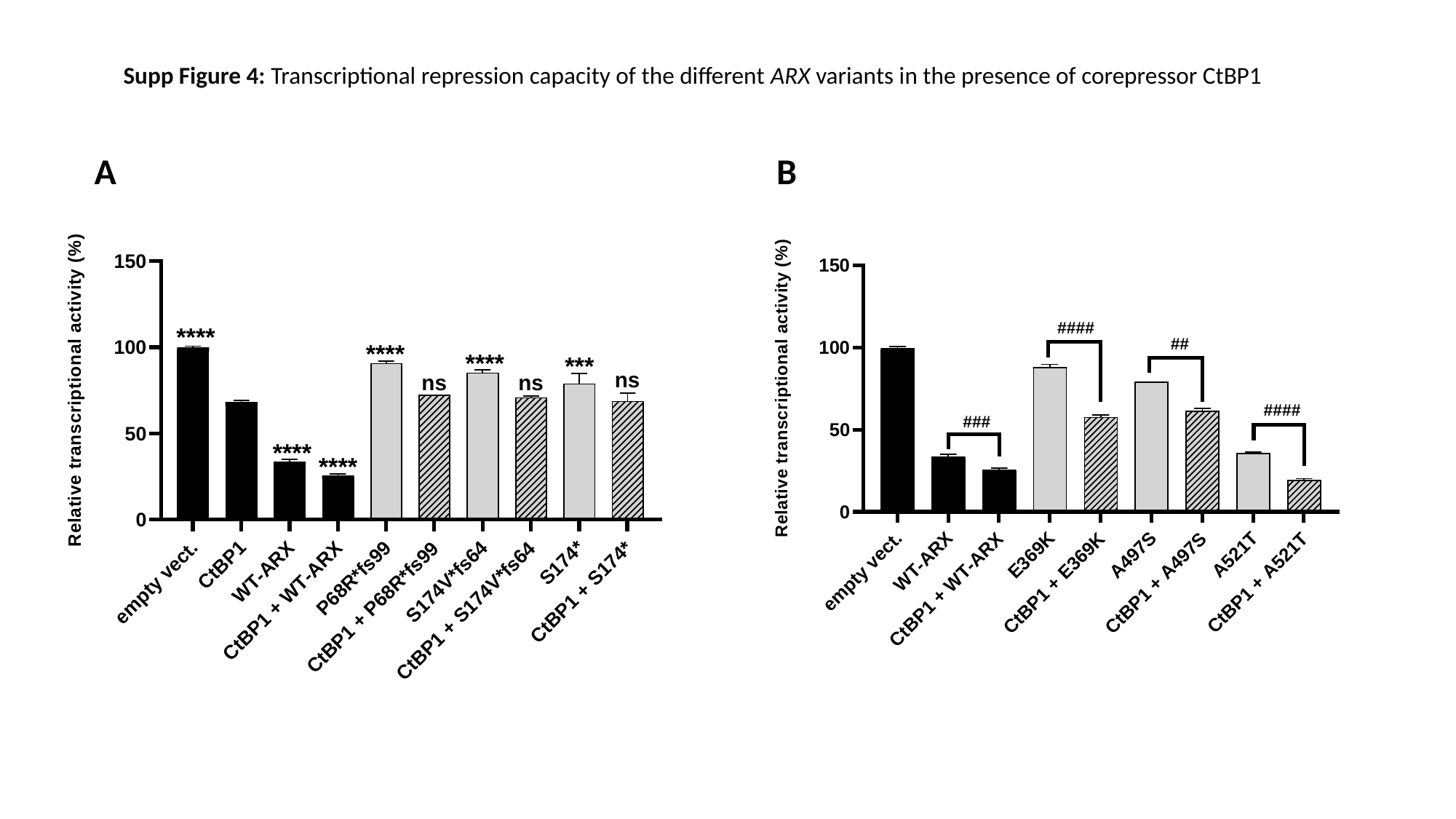

Supp Figure 4: Transcriptional repression capacity of the different ARX variants in the presence of corepressor CtBP1
B
A
####
##
####
###
